# Functional characterization of dynamic nascent RNA folding ensembles in real time

**DOI:** 10.1126/sciadv.aec4037

**Published:** 2026-03-20

**Authors:** Kavan Gor, Eva Maria Geissen, Olivier Duss

**Affiliations:** ^1^ Structural and Computational Biology Unit, European Molecular Biology Laboratory, 69117 Heidelberg, Germany.; ^2^ Molecular Systems Biology Unit, European Molecular Biology Laboratory, 69117 Heidelberg, Germany.; ^3^ Faculty of Biosciences, Collaboration for Joint PhD Degree between EMBL and Heidelberg University, 69117 Heidelberg, Germany.; ^4^ Data Science Centre, European Molecular Biology Laboratory, 69117 Heidelberg, Germany.

## Abstract

RNA structure starts forming cotranscriptionally as the nascent RNA emerges from the RNA polymerase and is dynamically modulated by cellular factors. How individual RNA conformations, out of an ensemble of RNA molecules, relate to function is not well understood. Here, developing multicolor single-molecule fluorescence microscopy experiments, we track in real time nascent RNA structure formation, functionally characterizing up to eight different types of RNA molecules. We find that ribosomal proteins, RNA modification enzymes or antisense oligonucleotides specifically modulate a subset of the RNA folding classes. For example, we provide direct evidence that increased local RNA accessibility at specific sites correlates with the chaperoning activity of ribosomal proteins during ribosome assembly. These experiments provide a general framework to study how dynamic RNA folding, and misfolding, relates to function.

## Teaser

Single-molecule imaging shows the role of various factors in selectively reshaping nascent RNA molecules to modulate function.

## INTRODUCTION

RNA structure is central to multiple cellular processes across the tree of life. The ability of RNA to form complex structures enables the binding of various cellular factors such as proteins, nucleic acids, ions, and small molecules, which can together undergo dynamic conformational changes to impact function (*1*–*4*). Despite the indispensable role of structured RNAs, the exact rules governing the process of RNA folding that leads to formation of complex functional structures remain elusive, mainly due to RNAs dynamic and flexible nature, often assuming multiple conformations (*5*, *6*). These properties also explain the lower performance of deep learning methods for RNA structure prediction in comparison to proteins (*7*–*10*).

Advances in computational methods to analyze high-throughput RNA structural mapping data have allowed the dissection of RNA structural heterogeneity by providing single-molecule snapshots of RNA structure in cellulo (*11*–*14*). However, these methods do not provide real-time dynamic information and, thus, make direct correlation of RNA structure to function difficult. Furthermore, the complex cellular environment complicates examining the specific effects of various factors on RNA structure formation, making it difficult to learn the biophysical principles of how RNA folds, because the majority of RNAs are expected to “fold correctly” in vivo.

In contrast, in vitro studies allow dissecting the effect of various factors affecting RNA folding, one at a time. For instance, folding intermediates of various RNAs have been previously studied by DNA oligo hybridization to specific sites of RNA, followed by RNase H cleavage, reporting on single-stranded RNA regions (*15*). A similar approach was used to study the role of RNA polymerase (RNAP) pausing in the cotranscriptional folding of the ribonuclease (RNase) P RNA, the signal recognition particle RNA, and the transfer-messenger RNA, thereby highlighting that the evolutionarily conserved pause sites enable formation of non-native labile RNA secondary structures that sequester the upstream regions of the RNA until the complementary strands are transcribed to form long-range helices (*16*). While these studies have provided an initial framework to assess the kinetics of RNA folding, they did not dissect the RNA folding heterogeneity nor provided real-time dynamic information.

In contrast, state-of-the-art single-molecule studies have probed in real-time dynamic RNA folding of small pretranscribed RNAs, such as riboswitches (*17*–*21*) or segments of ribosomal RNA (rRNA) (*2*, *22*), investigated the effect of transcription on RNA folding (*23*–*29*), and used protein binding as an indirect readout for RNA folding efficiency (*24*, *28*) but have not directly correlated dynamic nascent RNA structure formation with function at the single RNA level. For example, Chauvier *et al.* (*23*) have used the concept of dynamic RNA structure probing [single-molecule kinetic analysis of RNA transient structure (SiM-KARTS)] using short DNA probe binding to the RNA to study the folding kinetics of the fluorine riboswitch. Their assays highlight the dynamic competition between the fluorine ligand and the NusA protein, which governs transcription regulation, but their approach does not track RNA structure and protein binding simultaneously. Furthermore, such studies have been performed only on relatively small RNA molecules, which effectively reduce the probability of RNA molecules to misfold.

Large, highly structured RNAs like the 16*S* rRNA fold into complex secondary and tertiary structures cotranscriptionally, bind multiple ribosomal proteins (r-proteins) sequentially, and are chemically modified and processed simultaneously to eventually form ribosomes (*30*–*33*). Early studies have investigated the vectorial (5′ to 3′) folding of rRNA and the sequential as well as cooperative nature of r-protein assembly (*34*–*39*). Technological advancements allowed probing reaction kinetics that highlighted the presence of parallel and heterogeneous folding pathways (*40*–*44*). While these studies used pretranscribed and prefolded RNA and correlated the results into a cotranscriptional folding model, recent studies using single-molecule fluorescence microscopy on nascent RNA have directly visualized the cotranscriptional aspect of r-protein assembly (*24*, *25*, *28*, *45*). These studies have pointed out the heterogeneity of the assembly process and associated it with RNA misfolding, but have only partially investigated the folding dynamics of the RNA directly (see Supplementary Text) (*25*).

Here, using multicolor single-molecule fluorescence microscopy, we are simultaneously tracking transcription elongation of the 3′ domain of the 16*S* rRNA, are probing the accessibilities of RNA sites on the nascent RNA, which includes cotranscriptional as well as posttranscriptional RNA structure relaxation, and are assessing the binding of a protein as a readout for functional RNA structure formation. Using up to five different fluorescent dyes within a single experiment, we can simultaneously study the dynamics of up to eight different nascent RNA molecule types (folding classes), limited by the number of probes that we can simultaneously track. We see how various factors, such as RNA modification enzymes, r-proteins or antisense oligonucleotides (ASOs), specifically affect a subset of these folding classes. Our assays illustrate a variety of misfolding behaviors and dissect the heterogeneity of the structural ensemble during ribosome assembly. Overall, we provide an unprecedented view of the complex and understudied process of dynamic RNA folding and misfolding.

## RESULTS

### Short DNA probes can be used to detect dynamic RNA accessibility profiles

**Fig. 1.**
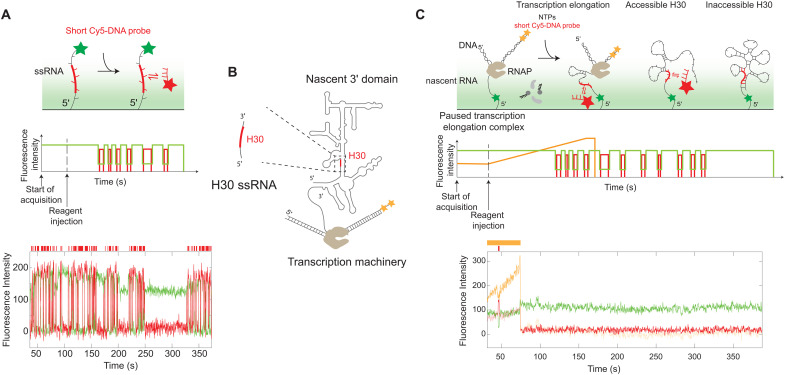
Concept of single-molecule dynamic RNA structure probing assay. (**A**) Schematic of experimental setup to probe single-stranded RNA (ssRNA) (top), idealized trace (center), and an example smoothed trace (bottom) showing the binding and unbinding of the probe (red, acceptor Cy5 dye) to the ssRNA (green, donor Cy3 dye) detected using FRET. (**B**) Schematic of H30 ssRNA (left; used in A) and nascent 3′ domain transcribed by the transcription machinery (right; used in C). (**C**) Schematic of the experimental setup to simultaneously monitor transcription and RNA accessibility in real time (top), idealized trace (center), an example smoothed trace (bottom) showing transcription (yellow, Cy3.5 dye) and H30 probe binding (red, acceptor Cy5 dye; and green, donor Cy3 dye).

**Fig. 2.**
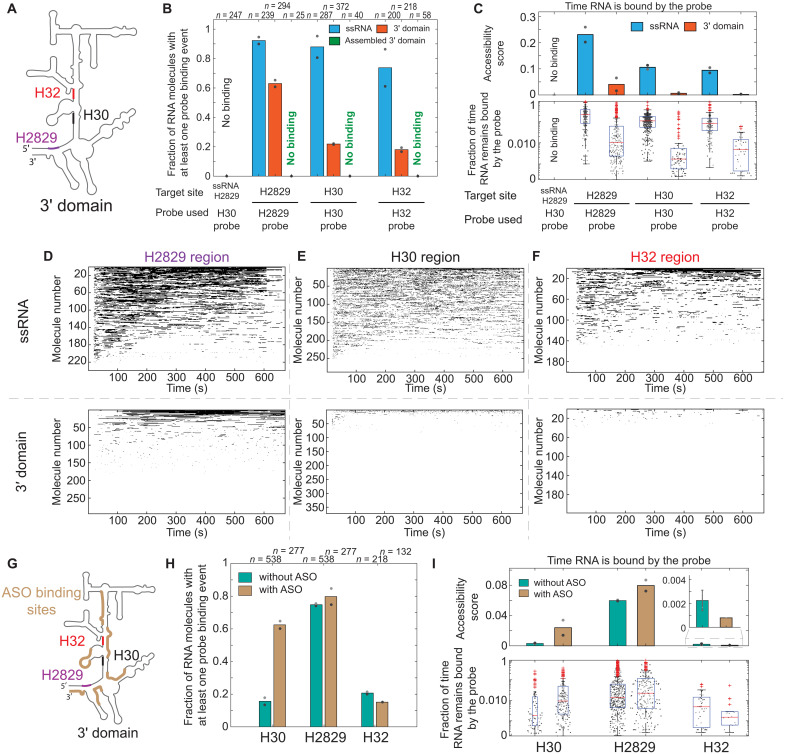
Monitoring RNA accessibility of helices in the 3′ domain of the 16*S* rRNA. (**A**) Schematic of the 3′ domain highlighting the different regions probed. (**B**) Fraction of RNA molecules accessible by DNA probes. (**C**) The accessibility score is the mean fraction of time the RNA molecules remain bound by the probe (top). The fraction of total experimental time the individual RNA molecules remain bound by the probe is represented as individual dots (bottom plots). (**D** to **F**) Rasterplot representation showing individual molecules as rows with black and white bars representing probe bound or unbound, respectively. (**G**) Schematic of the 3′ domain with the six ASO binding sites highlighted. (**H** and **I**) Effect of ASOs on the fraction of RNA molecules accessible by the DNA probes (H) and RNA accessibility score and the fraction time bound by probe for individual RNA molecules (I). The without ASO data [in (H) and (I)] is the same as presented in (B). [(B) and (H)] Number of molecules (*n*) used for analysis is indicated. Same number of molecules (*n*) were analyzed for (B) and (C) and (H) and (I). [(C) and (I)] The median and the quartiles in the overlayed boxplot are calculated by pooling the replicates. [(B), (C), (H), and (I)] Mean of two replicates is shown as bar, individual replicates shown as light and dark gray dots.

To provide dynamic structural information on single RNA molecules in real time, we used a previously established concept in which the transient binding of short DNA probes to specific RNA sites serves as a proxy for single-stranded RNA at these sites (*19*, *46*) (Fig. 1). As positive controls for RNAs with maximal probe accessibility, we first tracked the binding of short 7–nucleotide (nt)–long DNA probes to short single-stranded target RNAs (ssRNAs) that were immobilized to a glass surface for single-molecule imaging by total internal reflection fluorescence (TIRF) microscopy (Fig. 1A). Specific and transient binding of the short DNA probe to the ssRNA was detected by Förster resonance energy transfer (FRET) between the ssRNA, labeled with a donor dye (Cy3), and the DNA probe, labeled with an acceptor dye (Cy5, Cy5.5, or Cy7). We chose three different single-stranded RNAs comprising short stretches (7 nt) of the 3′ domain of the *Escherichia coli* 16*S* rRNA: (i) the linker region between helix 28 (H28) and H29 (residues 933 to 939; in the following referred as to H2829), which is part of the extended binding site for primary binding r-protein S7, (ii) the 5′-half of the tip of long-range H30 (950 to 956), which needs to form to stabilize the S7 binding site, and (iii) the 5′-half of H32 (986 to 992), a long-range helix, which is not part of the S7 binding site (Fig. 2A and fig. S1A) (*34*).

We find that for the specific targets, around 90% of the RNA molecules show repetitive DNA probe binding events, in agreement with a mostly single-stranded RNA structure (Fig. 2B). In contrast, we do not detect probe binding events to a nontarget RNA (H30 probe does not detectably bind to H2829 ssRNA) (Fig. 2B and Materials and Methods). The majority of the DNA probe accessible RNA molecules remain accessible during the entire 10 min experimental time, which we quantify by an RNA molecule–specific accessibility score (see Materials and Methods and Fig. 2C). The on-rate of the probe is linearly dependent on the concentration of the probe as expected for a single fully accessible RNA conformation (concentration independent on-rate: 1.8 × 10^−6^ M^−1^ s^−1^ for H2829; fig. S1B) (*46*–*48*). At 200 nM probe concentrations, the median DNA probe arrival times are 3.2, 11.5, and 10.2 s and the median bound-lifetimes of individual binding events of our probes are 1.6, 5.7, and 11.5 s for H30, H32, and H2829, respectively. Kinetic analysis of the bound lifetimes indicates single exponential behavior (fig. S1C). Overall, this setup allows us to track specific RNA accessibilities in a timescale of a few seconds resolution.

### Site-specific RNA accessibilities during folding of the nascent 16*S* rRNA 3′ domain

Next, we sought to investigate how these various RNA segments engage in RNA structure formation in context of an actively transcribing RNA which is highly structured once entirely transcribed and natively folded. We chose the 3′ domain of the 16*S* rRNA in *E. coli* (Fig. 1B) as it was previously well characterized by various biophysical and biochemical approaches. For example, we previously tracked the cotranscriptional formation of the long-range H28 by donor and acceptor dyes located at the 5′-end and 3′-end of the 3′ domain (*25*). While these data allowed us to determine whether a single RNA molecule had H28 formed or not, technical limitations (see Supplementary Text) prevented us from determining at what time point this helix was formed and from providing dynamic site-specific RNA folding information on the rest of the >440-nt-long 3′ domain intervening RNA.

To this end, we immobilized a stalled transcription elongation complex (TEC) via the 5′-end of the nascent RNA (see Materials and Methods) to the surface for multicolor single-molecule imaging (Fig. 1C). At the start of the reaction, we delivered a short DNA probe and nucleotide triphosphates (NTPs) to the immobilized stalled complex, which triggered transcription elongation of the 3′ domain rRNA (Fig. 1C). As the transcription progresses, the Cy3.5-labeled 3′-end of the DNA template approaches the imaging surface causing an exponential increase in fluorescent signal intensity, which reports on transcription elongation (*24*, *25*). Our labeling strategy allowed us to simultaneously track active transcription elongation, determine at what time point the full-length 3′ domain RNA has completed transcription (complete signal loss of both Cy3.5 dyes upon dissociation of the DNA template from the surface as opposed to stepwise photobleaching of both Cy3.5 dyes) and when the specific RNA sites are accessible during transcription and following dissociation of the transcription machinery of the nascent RNA (fig. S1D), thus providing site-specific and dynamic RNA structure information of single nascent RNA molecules.

Kinetic characterization of probe binding to the 3′ domain shows single-exponential behavior for the regions probed, except for the H2829 region, which has an additional minor population (fig. S1C and Supplementary Text). RNA mutants disrupting the probe binding target sites show that the probes bind specifically, as we do not detect off-target binding to any part of the entire 3′ domain (fig. S2, A to E). To ensure that our FRET-based probe detection approach captures all the probe binding events in the different possible RNA conformational states, we repeated our experiments with a Cy3B-Cy5 FRET pair to extend the accessible FRET range (Cy3B-Cy5: *R*_0_ = 71.9 Å; Cy3-Cy5: *R*_0_ = 51 Å; https://fpbase.org/fret/) and find comparable fractions of molecules with probe binding for all three measured sites (fig. S2F and Supplementary Text). We also verified that the transient binding of our probes does not detectably affect cotranscriptional RNA folding: The fraction of RNA molecules that bind r-protein S7 (proxy for successfully folded RNA molecules; see below) is not significantly affected, as well as the time from transcription initiation till appearance of the first S7 binding event is unaffected by the presence of the DNA probes targeting various RNA sites (fig. S1, E and F). Overall, all these controls demonstrate that we can robustly track nascent 3′ domain RNA structure formation using our short DNA probes.

In contrast to the short ssRNAs, for the entire 3′ domain rRNA, the fractions of nascent RNA molecules that bind the probe substantially decrease for all the sites. They decrease from 80 to 90% to ~20% for H30 and H32. In contrast, still ~60% of the RNA molecules remain accessible to the H2829 junction DNA probe, suggesting that local accessibility dynamics vary between different sites on the naked rRNA (Fig. 2, A, B, and D to F). For the fully assembled 3′ domain, identified by bound tertiary r-protein S3-Cy5.5, we did not observe any probe binding (Fig. 2B and fig. S3), in line with the expectation that these sites are completely protected in the natively assembled 3′ domain (*34*, *37*). When comparing short ssRNAs versus the nascent 3′ domain rRNA, the median RNA molecule-specific accessibility score reduced for all the sites by one to two orders of magnitude (Fig. 2C), in agreement with global cotranscriptional RNA structure formation (*34*, *37*, *40*, *42*, *49*). We additionally observe high variation in this accessibility score between molecules, which we attribute to the heterogeneous conformational nature of RNA molecules (see Supplementary Text).

Next, we designed ASOs that can bind to different regions of the 3′ domain with the aim to perturb cotranscriptional RNA folding dynamics (Fig. 2G). In contrast to the short DNA probes (7 nt) that were designed to bind transiently to the nascent RNA, the DNA ASOs were designed to remain stably bound to the nascent RNA and therefore, had a length of 18 nt (*50*). Upon simultaneous addition of six ASOs covering several regions across the entire 3′ domain rRNA, accessibility of the H30 site increases from ~18 to ~62%. In contrast, for the H2829 and H32 sites, we do not detect substantial accessibility increases despite the presence of the six ASOs (Fig. 2, H and I, and fig. S4; see Supplementary Text).

Overall, probing the accessibility of different sites across the nascently transcribed 16*S* rRNA 3′ domain, our data show that sites, which are predicted to be inaccessible in the natively folded 16*S* rRNA structure, are transiently accessible in a subpopulation of RNA molecules, at various degrees for the different sites. This raises the question whether the subset of probe-accessible RNA molecules may constitute misfolded RNA molecules.

### Correlating rRNA accessibility with r-protein binding as functional readout

**Fig. 3.**
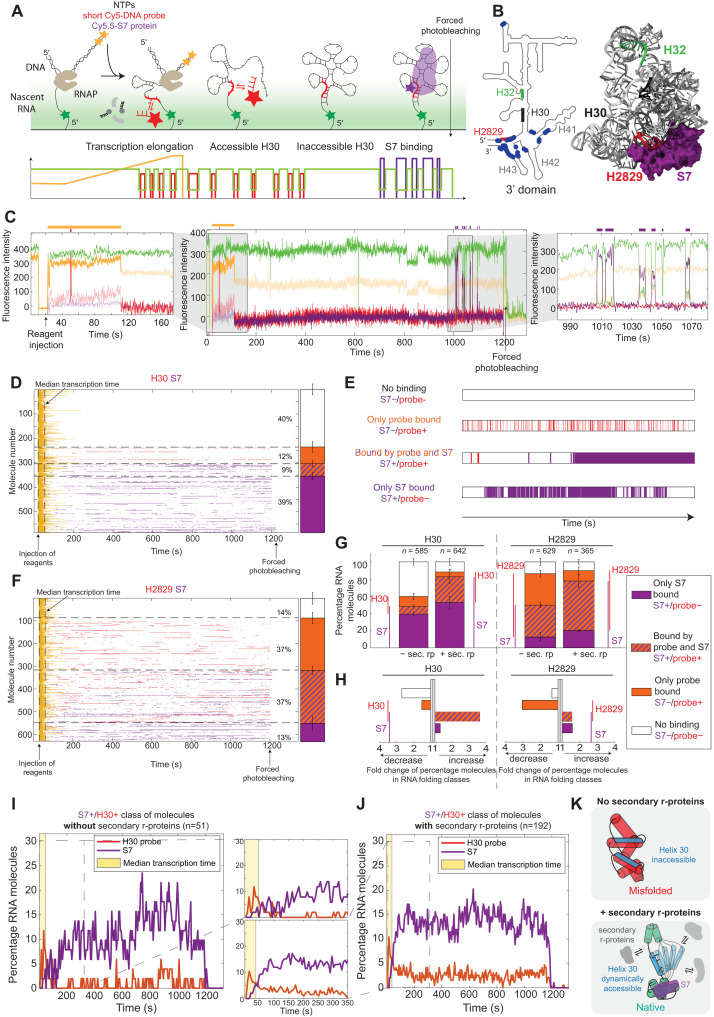
Assigning function to individual RNA folding classes. (**A**) Schematic of the experimental setup. (**B**) Secondary (left) and three-dimensional structure (right) of the 3′ domain highlighting the different regions probed: H2829 (red), H30 (black), H32 (green) and contact sites of S7 to RNA (blue circle) (PDB accession code: 4V9P). (**C**) Idealized trace (top) and an example smoothed trace (bottom) showing transcription (yellow, Cy3.5 dye), FRET donor (green, Cy3 dye), short DNA probe that is probing RNA accessibility (red, Cy5 dye), and binding of r-protein S7 (purple, Cy5.5 dye). (**D** and **F**) Rasterplots depicting the individual molecules as rows with transcription (yellow), DNA probe (red) and S7 (purple) binding events shown as colored bars for H30 (D) and H2829 (F) sites, respectively. The sorted rasterplots show four RNA folding classes: No binding events as S7−/probe− class (white); only probe bound class as S7−/probe+ (orange); S7 bound and probe bound class as S7+/probe+ (stripes of orange and purple); only S7 bound class as S7+/probe− (purple). (**E**) A simplified example trace from each class is shown. (**G** and **H**) Percentage of molecules in RNA folding classes (G) and relative fold change (H) upon addition of secondary r-proteins (sec. rp). (**I** and **J**) Time-dependent analysis of the S7+/H30+ class of molecules in absence (I) and presence (J) of the secondary r-proteins. The region highlighted in yellow represents the median time RNA is associated to the transcription machinery. (**K**) Model representing increased H30 accessibility in presence of secondary r-proteins. [(D) and (F) to (J)] Data represented by pooling three replicates, error bars show weighted SDs, number of molecules analyzed (n) is shown.

To functionally characterize the heterogenous pool of nascent rRNA conformations at the single RNA level, we repeated our site-specific RNA accessibility probing experiments in presence of r-protein S7 (Fig. 3A). The binding site of primary r-protein S7 extends across and covers the three-way junction of H28/H29/H43 and the four-way junction of H29/H30/H41/H42 (Fig. 3B and fig. S5A). Thus, successful binding of S7 to the nascent RNA serves as a convenient proxy for assessing the native folding state of the entire lower part of the 3′ domain (*25*).

To simultaneously track site-specific RNA accessibility (DNA probe binding) and functional RNA structure formation (S7 binding to the nascent RNA), we added 20 nM Cy5.5-labeled S7 in addition to Cy5-DNA probe and NTPs to our nascent RNA folding assay (Fig. 3A,C). FRET-based readout allows us to detect only specific S7 binding events, which we additionally verified by mutating the rRNA at its S7 binding site: This abolished the S7 binding almost completely (fig. S5, B to D), in line with previous reports that used an S7 protein mutant to show the same (*25*). The median arrival time of S7 to wild-type 3′ domain RNA was ~12 s, and the bound lifetime could be classified into two average S7-bound phases: short-lived (~1 s) and longer-lived (~10 to 20 s), as described previously and summarized in table S1 (fig. S5E).

To investigate the dynamic RNA conformational heterogeneity, we classified all the RNA molecules into four classes: Two S7 binding-competent classes S7+/probe− (no probe binding) and S7+/probe+ (with probe binding), which we assign as correctly folded and thus functional RNA molecules at least once during the experimental time, and two S7 binding-incompetent classes S7−/probe+ (with probe binding) and S7−/probe− (no probe binding), which we assign as misfolded and thus nonfunctional RNA molecules during the entire experimental time (Fig. 3, D, E, and F, and figs. S6 and S7).

Under our experimental conditions, ~50% of the molecules could bind S7, similar for both the tested probes targeting H2829 (~49%) and H30 (~48%) regions on the rRNA, which is in agreement with our probes not affecting RNA folding. For the H30 region, only a minority (~18%) of the S7 binding-competent RNA molecules also show probe binding, in agreement with successful H30 formation required for S7 to bind. In contrast, for the H2829 site, we see that ~75% of the S7 binding-competent molecules have the H2829 site accessibility during the experimental time (Fig. 3F). This finding was unexpected, considering that H2829 is at the center of the S7 binding site and includes several intermolecular interactions between H2829 and S7 (Fig. 3B and fig. S5A). While we do not see concomitant S7 and H2829 probe binding, this major class of RNA molecules transitions back and forth between S7 binding competent and H2829 accessible states multiple times during the 20 min experimental time, suggesting a dynamic equilibrium between a folded and slightly unfolded S7 binding site at this central region during this early stage of ribosome assembly (fig. S6). Mutations introduced to stabilize H2829 caused reduced fraction of molecule with H2829 accessibility, while mutations that decreased H2829 stability decreased the fraction of S7 binding molecules, effectively shifting the equilibrium (fig. S8).

Our data allow us not only to assign different RNA conformations into different nascent RNA folding classes but they also provide information on the progression of nascent RNA maturation from cotranscriptional RNA folding to posttranscriptional RNA relaxation following dissociation of the nascent RNA from the transcription machinery. We plotted the aggregated probe accessibility over time for the RNA folding class S7+/H30+ (see Materials and Methods). We find that in the first 50 s, which coincides with the median time that the nascent RNA is associated with the transcription machinery, there is substantially increased H30 accessibility till it subsequently decreases as soon as S7 starts to bind (Fig. 3, I and J, and figs. S7 and S9A). In contrast, for the S7+/H2829+ class, we do not detect increased probe binding to early nascent RNA but rather see probe binding during the entire experimental time (figs. S6 and S9C). This is in agreement with a dynamic interchange between two conformations, one with high H2829 linker accessibility and one with S7 binding, as described above.

### Secondary r-proteins guide folding by increasing local RNA accessibility

To understand how the RNA folding classes change during the course of ribosome assembly, we next investigated the effect of secondary r-proteins (S9, S13, and S19) on the cotranscriptional rRNA folding dynamics. Ribosome assembly is a sequential process in which secondary r-proteins, such as S9, S13, and S19, can only detectably bind after successful binding of primary ones, such as S7 (*31*, *39*, *51*). As expected, the fraction of S7-binding competent molecules increased from ~50 to ~80% in agreement with an RNA chaperoning function of the secondary r-proteins for the 3′ domain (Fig. 3, G and H, and fig. S9, D and E) (*25*). Furthermore, the median *K*d of S7 for the individual RNA molecules decreased for both S7+/probe− and S7+/probe+ classes by 1.5-/1.8-fold (for H2829 site) and 1.8-/1.2-fold (for H30 site), respectively (fig. S10, A to D). In agreement, the median S7 arrival times per molecule decreased (fig. S10, E and F) and S7 bound lifetimes (fig. S10, G and H) as well as longest S7 bound dwell time per RNA molecule (fig. S11) increased in presence of the secondary binding r-proteins, to a similar extent for all S7+ folding classes (S7+/probe− and S7+/probe+).

Apart from the expected increase in the fraction of S7-binding competent RNA molecules, unexpectedly, we also observed a substantial increase (~20 to ~36% of total molecules) for the fraction of molecules accessible at H30 upon addition of secondary r-proteins, while accessibility to H2829 stays high (~73 to ~69% of total molecules). By separately analyzing the four RNA folding classes, we find that mainly the subset of RNA molecules with accessible H30 is responsible for the increased fraction of S7 binding competent RNA molecules in presence of the secondary r-proteins (S7+/H30+ class: 3.6-fold increase; S7+/H30− class: 1.3-fold increase), while the fraction of S7-binding competent molecules increases independently of H2829 accessibility (both S7+/H2829− and S7+/H2829+: 1.5-fold increase) (Fig. 3, G and H, and fig. S9, D and E). Since H30 is surrounded by the binding sites of the secondary r-proteins (fig. S1A), we hypothesize that their transient binding could rescue stable H30-dependent RNA folding traps (see Supplementary Text) (*52*, *53*), thereby making the region around H30 more dynamic as experimentally detected by more transient DNA probe binding to this region (Fig. 3K). Overall, simultaneously tracking site-specific RNA accessibility and functionality of the same RNA molecules, we find how secondary r-proteins change the nascent RNA conformational landscape distribution, whereby increased conformational dynamics at specific sites correlate with increasing rRNA folding efficiency mediated by the secondary r-proteins.

### ASOs and assembly factors can specifically modulate a subset of RNA folding classes

**Fig. 4.**
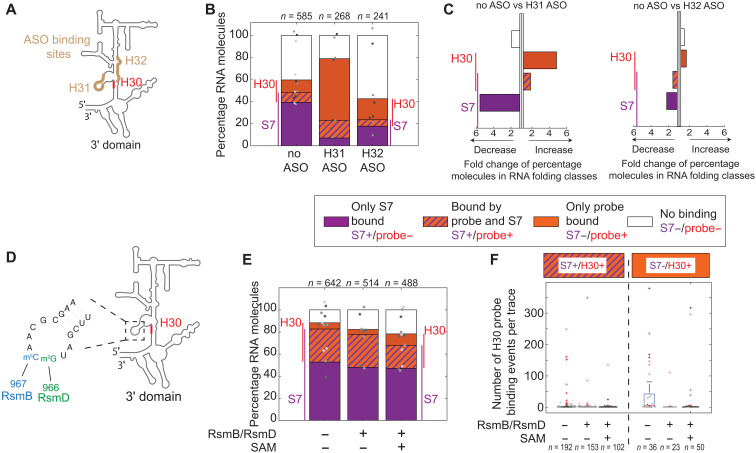
Modulating different RNA folding classes by assembly factors and ASOs. (**A**) Schematic of the 3′ domain with H30 site being probed (red) and the ASO target sites (brown). (**B** and **C**) Percentage of molecules in RNA folding classes (B) and relative fold change (C) in presence and absence of different individual ASOs. (**D**) Schematic of the 3′ domain with H30 site (red) and the corresponding nucleotides modified by RsmB (blue) and RsmD (green). (**E**) Percentage of molecules in RNA folding classes in absence or presence of RsmB/RsmD and/or SAM (always in presence of S9, S13, and S19). (**F**) Boxplot of number of H30 binding events per trace for S7+/H30+ (orange and purple stripes) and S7−/H30+ (orange) folding class in presence of secondary r-proteins with different combinations of RsmB/D and/or SAM. [(B), (E), and (F)] Number of molecules analyzed (*n*) is shown. Bars are calculated by pooling of 3, 2, and 2 [(B) and (C)] and 3, 2, and 3 [(E) and (F)] replicates respectively, shown as tones of gray dots. No ASO and no RsmB/RsmD/SAM condition is the same as presented in Fig. 3G.

Next, we tested if the different classes of RNA molecules can specifically be modulated by artificial and endogenous ligands. We first investigated the effect of ASOs binding to the upper part of the 3′ domain (intervening RNA flanking H30; Fig. 4A). This region lies outside of the S7 binding site and was shown not to be required for S7 binding, but when this region was present, it decreased the cotranscriptional rRNA folding efficiency by promoting rRNA misfolding (*25*). We therefore hypothesized that ASOs binding to this region would increase the fraction of S7 binding competent molecules by sequestering this upper region thereby reducing cotranscriptional rRNA misfolding of the extended S7 binding site (Fig. 4A). Unexpectedly, we find the opposite: A single ASO binding to H31 reduced the fraction of S7 binding competent RNA molecules 2.1-fold (~48 to ~23% decrease for total RNA molecules of combined S7+/H30− and S7+/H30+ classes). Notably, the effect is class specific: While the S7+/H30− class reduces substantially (5.6-fold decrease: ~40 to ~7%) there is, in contrast, an increase for the S7+/H30+ class upon ASO addition (1.8-fold increase: ~9 to ~16%) (Fig. 4, B and C, and fig. S12, A and B). Also, ASOs binding to other sites differentially affect the RNA folding classes, overall illustrating the complex redistribution of the nascent RNA conformational ensemble upon site-specific ASO binding, the effect on RNA structure difficult to predict without experiments (Supplementary Text).

Next, we wondered whether ribosome assembly factors that bind in the vicinity of the functionally important H30/H31 region could affect cotranscriptional rRNA folding. We chose the rRNA modification enzymes RsmB (methylating 967C to 967-m^5^C) and RsmD (methylating 966G to 966-m^2^G) presumably acting during early stages of 3′ domain assembly (Fig. 4D) (*54*, *55*). While they minimally affect the relative fraction of our four defined RNA folding classes (Fig. 4E and fig. S12, C, D, F, and G), they specifically and substantially reduce the number of H30 probe binding events (change of median number of binding events per trace: 5.5 to 1; and 75th percentile: 42.5 to 2 in absence and presence of RsmB/D, respectively; Fig. 4F) and showed a 3.2-fold reduction in the accessibility score (0.049 to 0.015; fig. S12E) for the S7−/H30+ class, an effect which is independent of the presence of the cofactor SAM required for the methylation (Fig. 4F and fig. S12, C to E). We hypothesize that by transiently sequestering the loop of H31, RsmB/D prevent the formation of the misfolded S7−/H30+ subclass with high probe accessibility. To test this hypothesis, we perturbed the RsmB/D binding site by replacing the H31 loop by an UUCG tetra-loop (fig. S13A). This minimally affected the RNA structural ensemble but reduced again the number of H30 probe binding events in molecules of the S7−/H30+ class substantially, being phenotypically equivalent to the presence of RsmB/D (fig. S13, B and C). These findings support a model in which loop H31 promotes the formation of a specific misfolded RNA class if not transiently sequestered by assembly factors or mutated. The effect of RsmB/D on only a small subset of the RNA molecules would be undetectable in bulk experiments, thus providing a first biophysical rational for the minimal growth phenotype under normal growth conditions for an RsmB/D double-deletion mutant (*56*). Overall, these data demonstrate how various nascent rRNA folding classes can specifically be modulated by artificial or natural ligands.

### Dynamic multisite RNA structure probing with functional readout

**Fig. 5.**
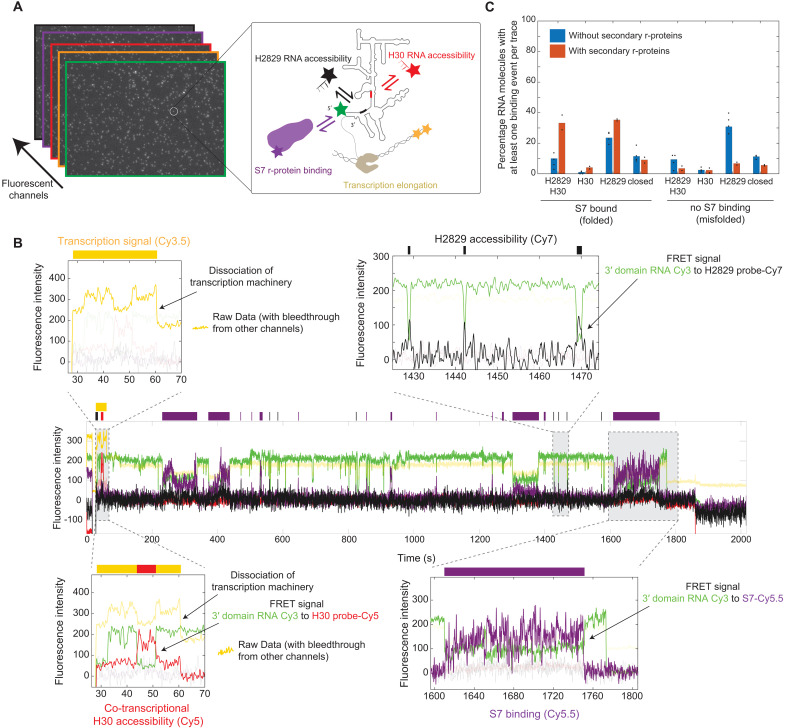
Multisite nascent RNA probing with functional readout. (**A**) Experimental setup for simultaneously probing transcription elongation, H2829 and H30 RNA site accessibilities, and the binding of r-protein S7, acquired from five fluorescent channels. (**B**) An example smoothed trace of the five-color data showing FRET donor (green, Cy3 dye), transcription signal (yellow, Cy3.5 dye), H30 probe (red, Cy5 dye), S7 r-protein (purple, Cy5.5 dye), and binding of the H2829 probe (black, Cy7 dye). Simplified representation of binding events is shown on top of the trace as bars. Insets zoom into specific regions highlighting the specific signals. (**C**) Percentage of RNA molecules within each of the eight folding classes in presence and absence of secondary r-proteins. Plotted by pooling four replicates – without and two replicates – with secondary r-proteins.

We showed that different RNA sites have different tendencies to remain dynamically accessible in functional RNA molecules. However, it is not clear whether different sites cooperate to be transiently accessible. To simultaneously track the dynamic accessibility of two sites and in addition, assign the functional state of the RNA molecules, we repeated our cotranscriptional RNA probing experiments by starting the reaction with the addition of NTPs, Cy7-H2829-DNA probe, Cy5-H30-DNA probe, and Cy5.5-S7 r-protein, requiring the simultaneous detection of five colors and the acquisition of >400 molecules for 30 min (Fig. 5, A and B, and fig. S14, A and B). Analogous to our above presented experiments, we classified the different RNA molecules into all possible accessibility and functional states, now resulting into 8 possible classes: ±H2829 accessible, ±H30 accessible, ±S7 binding-competent and combinations of these (fig. S14C, top rasterplot). We found molecules in all the eight classes, with the aggregated fraction of molecules in agreement with experiments in which only a single site was probed together with S7 functional readout (fig. S15A), using different labeling schemes (fig. S15, B and D) and for different replicates (fig. S15C), thereby validating our complex multicolor datasets and further confirming that various combinations of DNA probes used here do not detectably affect RNA folding dynamics.

Our data show that H2829 can be accessible independent of H30 accessibility, both for S7 binding competent and binding incompetent molecules (Fig. 5C and fig. S14C, bottom rasterplot). Repeating our experiments in presence of secondary r-proteins supports our finding that different sites can be accessible independently. We find that S7 binding competent classes, which are accessible by either H30 and/or H2829 probes increase in abundance in presence of secondary r-proteins, while the abundance of the S7 binding competent class, which is inaccessible to both DNA probes, does not substantially change in presence of secondary r-proteins (Fig. 5C and fig. S14C). Overall, these findings further support a model in which transient accessibilities at local RNA sites correlates with the ability of secondary r-proteins to chaperone nascent rRNA folding.

## DISCUSSION

**Fig. 6.**
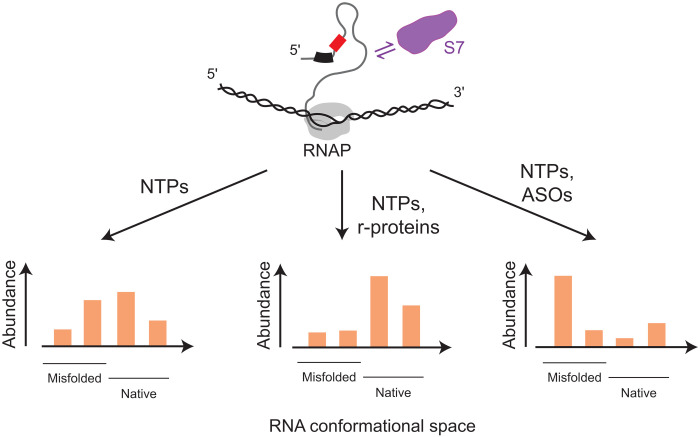
Ligands shape the nascent RNA conformational distribution landscape. The simplified model shows the effect of various factors on the distribution of the rRNA conformational landscape.

Using up to five-color single-molecule fluorescence imaging, we describe part of a dynamic RNA conformational ensemble sampled as nascent rRNA emerges from the RNAP and subsequently continues folding post-transcriptionally. Our data show how subsets of RNA conformations can differentially be modulated by various factors (Fig. 6). Unexpectedly, we find that RNA molecules with higher local dynamic accessibilities, constituting transient non-native structures, have a higher propensity to be chaperoned by secondary r-proteins and, thus, are overall more efficiently folded. In vivo, RNA is less structured and more dynamic, likely due to transient binding of RNA binding proteins and adenosine 5′-triphosphate (ATP)–dependent helicases (*57*). Overall, our data are consistent with a model in which dynamic RNA structure provides an advantage to form productive RNPs by minimizing RNA folding traps.

Previous biochemical and structural studies have investigated the heterogeneity in the rRNA folding and r-protein binding landscape using pretranscribed and prefolded RNA (*40*–*44*, *49*, *58*). Few studies have directly visualized the heterogeneity in r-protein binding to the nascently transcribed rRNA (*24*, *25*, *28*). Our experiments systematically dissect the cotranscriptional and posttranscriptional RNA structural heterogeneity at various levels. First, comparing individual RNA molecules of the structural ensemble, we show that different nascent RNA molecules probed at an individual site have high variability (>3 orders of magnitude) in their accessibility profiles (Fig. 2C). Second, comparing RNA accessibility across different sites further illustrates the heterogeneous behavior of the RNA. Third, simultaneously tracking rRNA accessibility and r-protein binding on the same nascent rRNA molecules revealed the presence of different RNA folding classes that would have been missed by only tracking RNA structure or protein binding at a time. Fourth, the molecules show heterogeneity in RNA folding and r-protein binding kinetics across molecules within a class as well as across different classes (Fig. 3). Last, simultaneously tracking the accessibility of two RNA sites with additional functional readout, we classified our dynamic structural ensemble into eight dynamic RNA folding classes, differing in both structural (local site accessibilities) and functional properties (the ability for a protein to bind the target) of single RNA molecules, which further highlights the nascent RNA structural heterogeneity (Fig. 5). However, the true structural diversity is much higher as we only detect information on a subset of all the sites at the same time. This structural diversity makes specific targeting of RNA structure much more challenging. Initial attempts to target RNA with small molecules were successful so far only for locally rigid structures such as GUC repeats, the 5′ splice site of SMN2 exon 7, or the ribosome, but more dynamic systems such as nascent rRNA present during ribosome assembly still await successful targeting (*59*–*62*). Successful targeting of RNA will require an ensemble description of RNA structure, as suggested by improved targeting of local RNA structural ensembles instead of a single static structure (*63*–*66*).

More promising is targeting of RNA with antisense oligonucleotides (*67*). We found a high variation in the fraction of RNA molecules that were accessible to our short hybridization probes at different local sites on the RNA. These findings provide a direct biophysical visualization and explanation of why certain sites in long mRNA molecules are more targetable by ASO than other sites (*68*–*74*).

While our data only allow us to classify the heterogenous pool of nascent RNA conformations into specific folding classes based on few local RNA accessibilities and the binding of S7, we can compare our dynamic and functional data to previous static RNA structure probing data. We found that the H30 probe binding is highly enriched during transcription, but binding is reduced as soon as S7 can bind (Fig. 3, I and J). Furthermore, destabilizing the region surrounding H30 resulted in a 5.6-fold reduction in S7 binding (Fig. 4 and fig. S13). Both findings suggest that H30 needs to be formed for S7 to bind. This is in agreement with footprinting data showing that H30 exhibits increased protection upon S7 binding despite lacking direct interactions between S7 and H30 (*34*), thus linking H30 formation with S7 binding.

Furthermore, hydroxyl radical footprinting of isolated native 16*S* rRNA in the presence of all 30S r-proteins showed that S7 forms immediate interactions with H43 but engages more than an order of magnitude slower with the H2829 junction (*40*). These data, together with our observations that post-transcriptionally, H2829 accessibility dynamically interchanges with S7 binding points to a model in which the H2829 junction is not stably preformed for S7 to engage immediately, but rather can switch between various open and closed conformations. These observations are also in agreement with S7 binding relatively late during 30S assembly despite being the primary binding protein of the 3′ domain (*75*, *76*).

In addition, we demonstrate that a single H31 ASO binding outside the S7 binding site strongly inhibited S7 binding and simultaneously increased accessibility of a neighboring RNA site (H30) in this misfolded (S7 binding incompetent) class of RNA molecules (sixfold decrease in S7+/H30− and fivefold increase in S7−/H30+ class), while no RNA folding class changed by >twofold for the H32 ASO (Fig. 4C). Footprinting data show that nucleotides bound by the H31 ASO strongly change reactivities upon addition of S7 and further upon addition of secondary r-protein S19 (*34*). Furthermore, H31 ASO encompassing nucleotides fold more slowly than most nucleotides on the H32 ASO binding site as measured by hydroxyl radical footprinting of the entire 16*S* rRNA in the presence of all 30S r-proteins (*40*). Last, H31 forms part of the core of the 3′ domain and requires assembly factor RimM for proper folding in vivo (*77*), highlighting its sensitivity to perturbations. Overall, these observations suggest that targeting H31 or the surrounding regions by an ASO has a large effect on RNA folding, as this helix is a critical checkpoint in 3′ domain folding.

Our structural and dynamic characterization of molecules into various RNA folding classes not only enables us to describe native (S7 binding competent) RNA molecules but also provides information on the pool of misfolded (S7 binding incompetent) RNA molecules. Research has focused on elucidating the functional native structures, because these are physiologically relevant and easier to study as they are usually present in a single well-defined conformation. In contrast, very little is known on the structural diversity and properties of misfolded RNA structures [(*25*, *28*, *78*–*80*) and this work]. Here, we also investigate the pool of RNA conformations that misfold during nascent RNA folding. For example, we observe a small class of molecules (S7−/H30+) that are kinetically trapped as they are accessible at H30 over the entire duration of the experiment and never refold into S7 binding competent conformations, irrespective of the presence of the r-protein chaperones (fig. S9B). Kinetic traps have been described for large RNAs like for the Tetrahymena ribozyme where incorrect topology leads to a long-lived misfolded state with the critical catalytic domain being mispositioned requiring extensive refolding for the misfolding to be resolved (*78*, *79*, *81*–*83*). The inability of some nascent rRNA molecules to rearrange over 30 min indicates that the RNA is highly structured, which may prevent “free ends” to initiate strand displacement, a mechanism for efficient rescue of RNA folding traps (*53*, *84*–*86*).

In contrast, the molecules in the S7+/H30+ class are initially accessible at H30 and then change to a S7 binding competent conformation. Furthermore, this effect is enhanced in the presence of r-protein chaperones. We associate this with the “preassociation” chaperoning mechanism of the r-proteins, where the transient association of the protein caused by nonspecific or a subset of specific interactions prevents the RNA from forming stable non-native interactions as a mechanism to rescue formation of folding traps (*52*). These weak non-native interactions could efficiently be rearranged via RNA strand displacement (*53*). We also observe high variation in accessibility across different sites. For example, unlike H30, the molecules of S7+/H2829+ class keep transitioning between a partially misfolded H2829 site and a S7 binding competent state. This local misfolding occurs at shorter timescales in contrast to the above-described stable kinetic RNA folding traps.

Overall, understanding mechanisms on how RNA can specifically misfold holds great promise for novel future strategies to target RNA: next-generation drugs could be designed to steer RNAs into, and specifically stabilize these misfolded and nonfunctional conformations to modulate cellular function.

## MATERIALS AND METHODS

### Experimental model

Plasmids for *E. coli* r-proteins S3 (WT and M129C), S7 [WT and S83C with a truncated C terminus (157 to 178)], S9, S10, S13, and S14 were a gift from the Williamson lab (*25*). The genes for *E. coli RsmB*, *RsmD* and S19 were cloned into a pESUMO vector backbone using Gibson assembly (Addgene ID: https://addgene.org/250650/, https://addgene.org/250651/, and https://addgene.org/250656/, respectively). Gene sequences were obtained from the ASKA collection plasmids (*87*).

### DNA templates and in vitro transcription

The DNA transcription templates were designed as described previously (*24*). In brief, the 3′-end of the DNA template was designed to have a single-stranded overhang where a DNA oligo could be hybridized. The single-stranded overhang was generated using autosticky polymerase chain reaction (PCR) using a gene fragment ordered from IDT (p0030_ab_fw and p0075_ab_bw). The PCR primers were designed to have a complementary sequence to the DNA template, an abasic site and the complementary sequence for hybridization to a labeled DNA oligo. PCR was performed using Phusion DNA polymerase as described in the manufacturer’s instructions. The presence of a single PCR product was verified on a 1% agarose gel, which was then purified using a Qiagen PCR purification kit following the instructions provided. If the PCR reaction contained side products, the correct product was purified from a 1% agarose gel using a Qiagen gel extraction kit, followed by buffer exchange into 10 mM tris HCl (pH 7.5) and 20 mM KCl using a 30 kDa molecular weight cutoff centrifugal filter (Amicon).

A 1.2× excess of DNA oligo labeled with two Cy3.5 fluorescently dyes (p0088_2xCy3.5, ordered from IDT) was used to hybridize to the single-stranded overhang of the DNA template by annealing at 68°C for 5 min and slow cooling at room temperature.

### Protein expression and purification

The proteins were purified in denaturing conditions as described earlier (*25*). In short, the proteins (S3, S7, S9, S10, S13, and S14) were expressed in *E. coli* [BL21(DE3) Gold strain] at 37°C in LB medium and the cells lysed in a microfluidizer. The resultant inclusion bodies were washed, then solubilized in 6 M guanidine chloride or 8 M urea (for r-protein S9 and S10) and dialyzed overnight in 8 M urea. The purification was then continued in denaturing conditions where samples were loaded on a SP HiTrap column (GE Healthcare) or a Ni–nitrilotriacetic acid (NTA) column (for r-proteins S9 and S10) followed by a SP column (for S9 r-protein). r-protein S10 was dialyzed overnight to cleave the SUMO tag at 4°C, followed by reverse Ni-NTA chromatography. The r-protein S10 was concentrated and dialyzed in the final buffer [50 mM tris HCl (pH 7.5), 400 mM KCl, 2 M urea, and 7 mM betamercaptoethanol] and frozen at −80°C. The S3, S7, S9, S13, and S14 r-proteins were dialyzed overnight in refolding buffer [20 mM tris HCl (pH 7.6), 20 mM NaCl, 0.5 mM EDTA, and 0.5 mM dithiothreitol (DTT)] at 4°C. The folded protein was then purified using a Heparin HP column (for r-protein S3, S7, and S14) followed by size exclusion chromatography on a Superdex 75 26/600 HiLoad gel filtration column (GE Healthcare) equilibrated with final sample buffer [20 mM tris HCl (pH 7.6), 100 mM NaCl, 0.5 mM EDTA, and 0.5 mM DTT]. r-protein S19 and RsmB and RsmD were expressed in LB medium at 37°C, cells lysed, and the lysate loaded on a Ni-NTA column (Cytiva), followed by overnight dialysis at 4°C to cleave the SUMO tag and reverse Ni-NTA chromatography. Proteins were further purified using a SP column (for r-proteins S19) or using size-exclusion chromatography on a Superdex 75 26/600 HiLoad gel filtration column (GE Healthcare) equilibrated with final sample buffer [50 mM tris HCl (pH 8), 200 mM NaCl, 0.5 mM EDTA, and 0.5 mM DTT] (for RsmB). The monomeric fractions were pooled and concentrated using an Amicon 3 to 10 kDa MWCO ultra centrifugal filter (Millipore) depending on the size of the protein and frozen at −80°C.

### Protein fluorescent dye labeling

The S7(S83C) variant with a truncated C terminus (157 to 178) and S3(M129C) variant were labeled using Cy5.5 sulfo-maleimide in denaturing conditions as previously described (*25*). In brief, 1 mg of r-protein was reduced using 10 mM DTT on ice for 2 hours in labeling buffer [100 mM Na_2_HPO_4_/KH_2_PO_4_ phosphate buffer (pH 7.0), 100 mM NaCl], followed by ammonium sulfate precipitation (70% w/v) and incubation on a rocker for 20 min on ice. The protein was pelleted (14000 rpm for 20 min) and washed with 70% ammonium sulfate in labeling buffer and again centrifuged at 14,000 rpm. The washed pellet was resuspended in labeling buffer containing 6 M urea. The r-protein was mixed with 1 mg of Cy5.5 sulfo-maleimide and incubated on a shaker (50 rpm) at room temperature for 30 min and the reaction stopped by adding 0.5% betamercaptoethanol. Excess dye was removed by purifying the reaction on a Nap5 column (GE Healthcare) equilibrated with labeling buffer containing 6 M urea. The resultant fluorescent fractions were visualized on a 4 to 20% SDS-PAGE using a Typhoon imager (Cytiva) and with Coomassie stain. The r-protein was refolded by diluting the sample 10-fold in sample buffer [20 mM tris HCl (pH 7.6), 20 mM NaCl, and 0.5 mM EDTA]. A second purification step was performed by passing the sample through a Heparin HP column at 4°C. The column was washed with 10 column volumes of sample buffer and the protein was eluted with 1 M NaCl in sample buffer. The fractions were visualized as mentioned above. The labeled fractions were pooled, aliquoted, and stored at −80°C.

### Preparation of stalled TEC for single-molecule imaging

The TEC for single-molecule imaging was generated as described earlier (*24*). To summarize, a paused TEC consisting of a DNA template, a single RNAP, and a nascent RNA of 50 nt (containing adapter sequences) was generated by incubating 25 nM DNA transcription template, 100 nM *E. coli* RNAP (in-house generated or purchased from NEB); 100 μM ACU trinucleotide (Dharmacon); 10 μM ATP, cytidine 5′-triphosphate (CTP), and uridine 5′-triphosphate (UTP; Jena biosciences); and 2 mM DTT in a buffer containing 50 mM tris HCl (pH 8.0), 14 mM MgCl_2_, 20 mM NaCl, 0.04 mM EDTA, bovine serum albumin (BSA; 40 μg/ml; nonacylated), and 0.01% Triton X-100 for 20 min at 37°C (*24*, *88*). Then, 20 nM of a preannealed double-stranded DNA oligo construct containing a biotin at the 5′-end and a single-stranded overhang at the 3′-end (p44 and p109-biotin) complementary to the adapter region on the nascent RNA was added to be annealed to the paused elongation complex along with heparin (1 mg/ml) to prevent transcription re-initiation. The mixture was incubated at 37°C for 20 min.

To perform the single-molecule assay, the microfluidic channel was functionalized and assembled as described earlier with a small adaption (*89*). Each channel was prepared such that it consisted of two inlets and two outlets. The channel and the inlet and outlet tubing were first washed with buffer [10 mM tris HCl (pH 8.0) and 50 mM NaCl] and incubated with neutravidin (0.1 mg/ml) for 5 min, followed by incubating the TEC for 10 min in imaging buffer [50 mM tris HCl (pH 7.5), 14 mM MgCl_2_, 20 mM NaCl, 0.04 mM EDTA, BSA (40 μg/ml; nonacylated), 0.01% Triton X-100, 2 mM spermidine, 1 mM putrescine, and 150 mM KCl].

### Probe design

The probes were designed such that they have only one site that is fully complementary (containing seven consecutive nucleotides) and do not have alternate target sites with more than four consecutive nucleotides complementary in the entire 3′ domain to minimize the detection of off-target probe binding (fig. S2).

### Single-molecule cotranscriptional RNA accessibility assay

To track DNA probe and protein binding during and following transcription, we had to hybridize a Cy3-DNA oligonucleotide to the 5′-end of the nascent RNA: After immobilizing the stalled TEC (ATP, CTP, and UTP stalled) to the imaging surface and washing away excess nucleotides, we walked the RNAP (10 min at 21°C in imaging buffer containing 10 μM ATP, CTP, guanosine 5′-triphosphate (GTP), and 100 nM p66-Cy3-FQ oligo or p153-Cy3B-FQ only when specified) such that the Cy3-oligo binding site on the nascent RNA becomes accessible for hybridization by the oligo. Last, the slide was washed with imaging buffer containing oxygen scavenging mix (OSC) consisting of 2.5 mM protocatechuic acid (PCA) and 250 mM protocatechuate-3,4-dioxygenase (PCD) to prevent photobleaching and 4 mM Trolox as triplet state quencher, ready for single-molecule imaging (*90*, *91*).

The assembled slide with immobilized molecules was used to focus molecules on the custom-built multicolor TIRF microscope. The parameters were setup (see instrumentation section) such that a 10-s laser dead time was encoded after 15 s from the start of the acquisition. The laser dead time was used to inject the reaction mixture (to prevent photobleaching of the immobilized molecules) containing 1 mM NTPs to re-initiate transcription elongation, 200 nM (or specified concentrations) of each of the labeled short DNA probes (to probe RNA accessibility, H2829 probe: p110Bcy5 or prKG058cy5.5 or prKG080cy7; H30 probe: prKG032cy5 or prKG057cy7; and H32 probe: prKG053cy5), and/or 20 nM S7-Cy5.5 (to provide functional readout), and/or 400 nM of each of the unlabeled secondary r-proteins, and/or 400 nM of each of the ASOs (H31: prKG074; H32: prKG073; H34: prKG072; H30-H41 junction: prKG075; H42-H29-H43 junction: prKG076; and H28: prKG077), and/or 400 nM of each of the unlabeled RsmB and RsmD proteins. The chase mix was prepared in the imaging buffer containing 0.25% Biolipidure 203, 0.25% Biolipidure 206, yeast total tRNA (0.5 mg/ml), and OSC (see above). To distinguish single molecules, as compared to multiple molecules present on a single diffraction limited spot, another reaction mixture was injected in the last 1% of the acquisition time. The second mixture was made in imaging buffer but containing only 0.25 mM PCA, 25 mM PCD, and 0.4 mM Trolox.

### Single-molecule assay to probe ssRNA/DNA accessibility

ssRNA/DNA labeled with Cy3 on one end and biotin on the other end and containing the region of interest of the 3′ domain was immobilized (ssRNA of H2829: p112A; H30: prKG047; H32: prKG078; and ssDNA: prKG081). The reaction mixture made in imaging buffer consisted of 0.25% Biolipidure 203, 0.25% Biolipidure 206, and yeast total tRNA (0.5 mg/ml; except for experiments in fig. S1B); 200 nM of the labeled probe and OSC mix were injected after the start of acquisition. The second injection mixture made in imaging buffer but containing only 0.25 mM PCA, 25 mM PCD, and 0.4 mM Trolox was injected in the last 1% of the acquisition time to induce photobleaching.

### Single-molecule assay to probe RNA accessibility in the preassembled 3′ domain

To assemble the 3′ domain, 400 nM of unlabeled r-proteins S7, S9, S13, S19, S10, and S14 and 400 nM of labeled S3(M129C)-Cy5.5 was added to the 3′ domain transcription reaction or to the 3′ domain rRNA that was pretranscribed and prefolded at 37°C. The reaction was incubated for 30 min. The preassembled complex was immobilized and used for probing RNA accessibility at 21°C as described above. Successful assembly of the 3′ domain was identified by S3(M129C)-Cy5.5 bound at the beginning of the experiment (Cy3-Cy5.5 FRET) as described previously (*25*).

### Instrumentation and data acquisition parameters

All the single-molecule experiments were acquired on a custom-built objective type (CFI SR HP Apochromat TIRF 100XC Oil) TIRF on an ECLIPSE Ti2-E inverted microscope (Nikon), built in collaboration with Cairn Research (https://cairn-research.co.uk/) and Ultimeyes (https://ultimeyes.eu/). Homogenous illumination over the full field of view and modulation of laser power was achieved using an iLAS modular scanning system interface (GATACA systems). The total fluorescent signal was split into their respective fluorescent channels based on the dye emission spectrum using a combination of bandpass and emission filters and the photons detected using three Prime95B sCMOS cameras (Teledyne Photometrics) with an exposure time of 100 ms at 21°C. All the experiments were acquired using an OBIS 532-nm LS 150-mW laser head with an output intensity of 0.2 kW cm^−2^ at the objective.

### Single-molecule data analysis

Single-molecule data from different cameras (two colors per camera) were split into stacks of individual fluorescent channels using the Metamorph software. Subsequently, the different fluorescent channels were organized into a single tile. The stacks were saved as Metamorph Multitiff files. The tile stacks were loaded into a modified version of the SPARTAN software (*92*) implemented in MATLAB. The localized molecules were visually inspected to check for drift over the duration of the experiment and drift corrected where necessary using an in-house written python script. The resultant drift-corrected files were processed with SPARTAN to perform channel alignment and registration as well as extraction of the individual single-molecule trajectories. The single-molecule trajectories were then further analyzed using MATLAB scripts as previously described (*24*, *25*, *93*, *94*).

The molecules were selected on the basis of specific criteria. For the cotranscriptional RNA accessibility experiments, the molecules were selected for two criteria: (i) molecules showing a characteristic gradual increase in fluorescent intensity of Cy3.5 (indication of transcription elongation) (*24*) followed by a single step drop in the fluorescent intensity (indicating DNA dissociation and thus dissociation of the transcription machinery) within 200 s; and (ii) molecules that have a Cy3 signal showing a single step photobleaching (indicating presence of only a single molecule at the diffraction limited spot) within the last 10 to 20% of the total experimental time. For experiments where only RNA was immobilized, the traces were selected on the basis of a single photobleaching step of the Cy3 oligo signal occurring during the last 10 to 20% of the total experimental time.

Specific binding of dye-labeled short DNA probes (Cy5, Cy5.5, and Cy7) and/or dye-labeled protein (Cy5.5) was assigned using an anticorrelated high FRET signal between the Cy3-donor dye (Cy3-labeled oligo attached to the 5′-end of the nascent RNA) and the acceptor dye (Cy5, Cy5.5, and/or Cy7). FRET efficiency was calculated as follows: *E*_FRET_ = *I*_A_/(*I*_A_ + *I*_D_), where *I*_A_ and *I*_D_ are the apparent fluorescent intensities of the acceptor and donor dyes, respectively. Because of substantial spectral cross-talk between neighboring channels, the bound state was assigned using a threshold set at the midpoint of the two FRET states with subsequent manual inspection of traces that showed nonspecific protein binding signal (causing a nonzero FRET efficiency baseline) as described previously in detail (*24*, *25*, *88*). In short, FRET efficiency was used as a guide to determine the specific binding; however, for traces with nonspecific protein binding, the anticorrelation of the individual donor and acceptor signal was used as a metric to distinguish the binding event from stickiness of protein to the surface resulting in high acceptor intensity but no simultaneous change in donor intensity.

### Accessibility score

The accessibility score provides an indirect value for describing the relative accessibility of a single RNA site under various experimental conditions. We calculate the accessibility score the following: First, for each single trace we determine the fraction of the time the RNA molecule is bound by the probe. The accessibility score is the mean of this value averaged over all the molecules of an experiment or a specific RNA folding class.

### Clustering

The rasterplots representing RNA accessibilities of individual sites in absence of r-proteins (Fig. 2, D to F) were generated by hierarchically clustering molecules using weighted pair group method with arithmetic mean and Spearman distance metric. The weighting of individual molecules was performed on the basis of the number of short DNA probe binding events.

### Time-dependent accessibility plots

After aligning the molecules postexperiment to the time of NTP delivery, the traces were binned into 5-s intervals and for each bin the percentage of molecules with (i) probe binding events and (ii) S7 binding events were determined and plotted as a line plot. The median time from the start of transcription to DNA template dissociation for the molecules present in the specific S7+/H30+ class was extracted and overlayed on the plot (Fig. 3, H and I).

### Kinetic and thermodynamic analysis of S7 binding from single-molecule data

The on-rate determination of DNA or protein ligands binding to longer RNAs in single-molecule experiments is challenging due to incomplete labeling efficiency of the ligands and importantly, the presence of multiple RNA conformational states observed in individual RNA molecules. On-rate distributions are therefore best described by multiexponential functions and thus challenging to interpret. Arrival times remain unaffected by these factors as they do not depend on any model.

Thus, deriving *K*d based on *k*off/*k*on is challenging due to challenges in determining the on-rate. Therefore, we determine the apparent *K*d from single RNA molecule binding to S7, assuming equilibrium of each RNA conformation, by using the fraction of bound RNA according to the below formula.

$$Kd,\text{sm}=\frac{1-Y}{Y}\;\left[P\right]$$ (1)

[*P*] describes the concentration of free protein. *Y* denotes the fraction of bound RNA [*Y* = *t*_PR_/(*t*_PR_ + *t*_R_) with *t*_PR_ denoting the total time when RNA (R) is complexed with protein (*P*), and *t*_R_ denoting the total time when the RNA is free]. Because the number of immobilized RNA molecules on the glass surface for single-molecule experiments is much lower than the total number of protein molecules added to the imaging solution, [*P*] ≈ [*P*]_0_ with [*P*]_0_ being the total labeled concentration of the added protein in our single-molecule experiments. This was used to derive the *K*d for individual molecules.

To determine the bulk *K*d across an entire class of RNA molecules, the molecules of the individual class were combined into a single molecule. The resultant bound time and unbound time were used to determine bulk *K*d using the above formula. The bulk *K*d was used to determine the fold change across different conditions. The data were evaluated between 200 s till donor photobleaching to calculate *t*_PR_ and *t*_R_ for both the above-mentioned *K*d analysis.

## Supplementary Materials

### This PDF file includes:

Supplementary Text Figs. S1 to S15 Tables S1 and S2 Materials References
