# Supplementary material for: Functional characterization of dynamic nascent RNA folding ensembles in real time
Source: Sci Adv. 2026 Mar 20;12(12):eaec4037. doi: 10.1126/sciadv.aec4037 (PMC13004024; doi:10.1126/sciadv.aec4037)
Supplement: Supplementary file 1 — Supplementary Text Figs. S1 to S15 Tables S1 and S2 Materials References [file sciadv.aec4037_sm.pdf]

Science Advances
AAAS

Supplementary Materials for

Functional characterization of dynamic nascent RNA folding ensembles in real time

Kavan Gor *et al.*

Corresponding author: Olivier Duss, olivier.duss@embl.de

Sci. Adv. **12**, eaec4037 (2026)
DOI: 10.1126/sciadv.aec4037

This PDF file includes:

Supplementary Text
Figs. S1 to S15
Tables S1 and S2
Materials
References

Supplementary Text

Comparing to existing single-molecule FRET approaches to monitor nascent RNA folding:
Previously (*25*), the formation of a long-range RNA helix, H28 in the 3'domain of the 16S rRNA, was tracked for a nascent RNA emerging from the RNAP. While this allowed determining which RNA molecules had H28 formed, it did not allow determining at what timepoint H28 formation happens, as explained in the following: Successful formation of long-range H28 was determined by detecting a Cy3/Cy3.5 FRET signal between Cy3 and Cy3.5 DNA oligonucleotides that were hybridized to artificial 19-22nts long sequences located at the 5'-end and 3'-end of the nascent RNA during transcription. However, the binding of the labelled DNA probes to the nascent RNA is very inefficient due to formation of secondary structure in the artificial probe binding region. This results into DNA oligonucleotide arrival times of dozens of seconds and thus, prevents determining the exact timepoints at which H28 forms and thus, does not allow for real-time tracking of RNA structure. Furthermore, this approach cannot be adapted for internal RNA structure but is limited to tracking the formation of long-range helices for which the RNA 5'-end and 3'-end base-pair.

Kinetic analysis of the probe binding to target sites:
In order to access the residence times of the probes, we fitted the bound dwell time distributions to single and double exponential functions. Comparing the fits of probe binding to the ssRNA and the 3'domain we observe that H30 and H32 show mostly single-exponential behavior as expected (fig. S1C). There is a slight deviation from perfect single-exponential behavior for H32 ssRNA, which is likely explained by some remaining RNA structure even in the small "ssRNA" construct, because probing of a ssDNA with identical sequence shows pure single-exponential behavior. Similarly, the on-rates of the probes to H32 ssRNA exhibited a double-exponential behavior, which transitioned to single-exponential behavior for H32 ssDNA. H2829 probe binding dwell times to H2829 ssRNA follow a single-exponential kinetics; however, the dwell times to the 3' domain cannot be explained by a single-exponential fit. One explanation could be that for a fraction of the binding events, the H2829 target site is only partially accessible (5 or 6 out of 7 nucleotides) for probe binding, resulting in faster dissociation of the probe. Another possibility could be that the complementary RNA strand during local RNA folding could push away the DNA probe (reducing its bound lifetime) by strand displacement in agreement with our data showing dynamic opening and closing of the H2829 site (transitions between S7 and H2829). We also note that for sites H30 and H32, for which we see only a single exponential behavior, the off-rate is faster for the 3'domain compared to the isolated ssRNA constructs. We hypothesize that RNA conformational dynamics, such as local strand displacement, or incomplete accessibility of the probe for its 7 nucleotides binding site (i.e. only 6 nucleotides full complementarity), lead to generally faster DNA probe dissociation in the 3'domain RNA versus their isolated binding sites.

Our FRET-based probe detection approach detects all the probe binding events to the 3'domain rRNA:
rRNA folds and compacts instantly in presence of Mg^{+2} (*2*) and therefore will never be fully unstructured and linearized, even if misfolded. The experimentally determined hydrodynamic radius of the entire 16S rRNA is 117 Å in 20 mM Mg^{+2} measured at 25 °C (*96*). The hydrodynamic radius of the isolated 3'domain is not experimentally characterized, but we have calculated the radius of the 3'domain using following empirical formula published previously (*97*).

$$R_H = 5 \times 10^{-10} \times N^{0.38} \qquad -(2)$$

R_H is the hydrodynamic radius, and N is the number of nucleotides.

The 475 nucleotides of the 3' major domain give a hydrodynamic radius of 52 Å. With a maximally possible separation of the Cy3-Cy5 FRET pair (R_0 = 51 Å; https://www.fpbase.org/fret/) on the hydrodynamic sphere of 104 Å (2x 52 Å), the FRET efficiency would drop to E= 0.014 based on the following formula:

$$E = 1/(1 + (R/R_0)^6) \qquad -(3)$$

E is FRET efficiency, R_0 is the Förster radius and R is the distance between donor and acceptor. In order to verify that all probe binding events are within FRET distance even in the unlikely events that the donor-acceptor pair is at the maximally possible separation, we repeated our experiments with the Cy3B-Cy5 dye combination (R_0 = 71.9 Å) which would give a FRET efficiency E=0.1 at 104 Å and thus, should be detected. Comparing our data for the Cy3-Cy5 and Cy3B-Cy5 FRET pairs shows an almost identical fraction of RNA molecule with probe binding for all three sites (fig. S2F). Thus, these experiments strongly support that we detect all probe binding events with our FRET-based detection approach.

Fraction of time the RNA remains bound by probe:

The heterogeneity observed in "fraction of time the RNA remains bound by probe" in the 3'domain can be explained by a heterogeneous population of RNA molecules, in which individual molecules transition between various RNA conformations. For example, the most heterogeneous H2829 region, in which "the fraction of time RNA remains bound by probe" varies by more than three orders of magnitude between different RNA molecules, is consistent with the idea that RNA molecules transition between H2829 accessible conformations (during which the probe can bind) and H2829 inaccessible conformations (in which S7 binds but not the probe). Thus, RNA molecules with little H2829 accessibility (e.g. having a single probe binding event) will have a much smaller "fraction of time the RNA remains bound by probe" than an RNA which is constantly accessible by the probe (which will have multiple probe binding events during the experimental time).

Increased H30 accessibility caused by secondary r-proteins:

In our previous work on the 3'domain assembly (*25*), and parallel work from the Woodson lab on the 5'domain assembly (*28*), we found that secondary r-proteins can chaperone nascent 3'domain rRNA folding by transiently interacting with the nascent RNA before the primary r-protein S7 is bound. At this stage, the binding sites for the secondary r-proteins are not created yet or only partially created, because secondary proteins can only stably bind to rRNA once the primary binding r-proteins are bound (*38, 98*). We believe that we cannot distinguish whether conformational selection or induced fit is responsible for the chaperoning effect mediated by the secondary r-proteins. The H30 accessible conformations belong to at least two types of RNA structures: 1) The S7+/H30+ class, which is increased in presence of secondary binding r-proteins, could represent a native assembly intermediate in which the H30 transiently opens (the H30 probe can bind) and closes (it needs to be closed when S7 binds). 2) The S7-/H30+ class, which does not increase in presence of secondary binding r-proteins, is likely non-native as it is kinetically trapped in an S7-binding incompetent conformation for at least 20-30 minutes (experimental time). From our data, we can tell that in the presence of secondary r-proteins, we see more molecules in the dynamic S7+/H30+ class (dynamic because it changes between H30 accessible and S7 binding competent conformations). However, whether this shift in population arises from conformational selection or induced fit mediated by the secondary r-

proteins, a decision which is likely made as soon as the nascent binding sites emerge from the RNAP, cannot be distinguished from our data.

SpikeTrain analysis is not applicable to our system:

We originally considered using the SpikeTrain analysis, but were unsuccessful due to our much more complicated system. Walter and co-worker's SpikeTrain analysis was facilitated by their much simpler system with a smaller RNA molecule, which is present mainly in two conformations, and thus, allowed a simple interpretation of the data when the molecules switch from conformation 1 to conformation 2. In our case, the nascent RNA molecules are very heterogeneous and will switch between multiple different conformations. While our probe-only binding data (Fig. 2) may suggest that the RNA exists in two well-defined conformations, our more complex experiments, including, in addition, a protein (Fig. 3), show that this is not the case and that there are at least 4 conformational classes. Finally, even further extending our system by tracking accessibility of two sites with oligo binding and using a protein to assess the fold of the S7 binding site, we see that we have at least 8 conformational classes (Fig. 5). Therefore, using spike-train analysis on such data, which would report on the transition between two states, would be wrong data interpretation. Alternatively, the determination of the number of states from such heterogeneous data is extremely challenging and would require at least 1 order of magnitude more data (which is currently not feasible for such complex multi-color single-molecule experiments). Furthermore, the frequency of binding of our probe (to our much more structured rRNA as used in the study from Walter and co-workers) is too small to reliably track exact structural transitions. Thus, SpikeTrain analysis is not applicable to our system.

The effect of ASOs on the RNA structural ensemble is difficult to predict:

Generally, none of the 6 ASOs bind directly to the oligo binding sites and thus, ASO binding exhibits indirect effects on modulating oligo accessibilities. Knowing that our RNA structural ensembles contain a large fraction of non-native conformations of unknown global structure, we cannot make good predictions on how multiple ASOs will affect RNA structure based on the native secondary structure (Fig. 2G). Furthermore, our data consist of a very heterogeneous set of nascent RNA conformations, each conformation differently affected by an ASO. Therefore, it is very difficult to predict the outcomes of ASO addition, and thus, we can only speculate on why the 6 ASOs do not affect the accessibility of H2829 and H32 but do so for H30 (Fig. 2H).

Oligo site accessibilities can be affected by either local structure (e.g., hairpin formation sequestering the probe binding site) or global RNA structure formation (e.g., formation of long-range helices like in the native H30 or H32 helices). H2829 is highly accessible in the absence of the 6 ASOs and remains highly accessible in the presence of all tested perturbations (see e.g. S8D,F and 3G), suggesting that the oligo binding site is not prone to sequestration by local RNA structure. Furthermore, we would expect that the binding of the H28 and H42 ASOs, which bind opposite to the H2829 oligo binding site in the native structure, would rather make H2829 more accessible, and our data is in agreement with this. In contrast, the H32 oligo site shows generally very low oligo accessibility, and we speculate that this is due to a high propensity to form local RNA structure around this site. Thus, even when ASOs perturb the rest of the RNA structure, the H32 oligo site will remain sequestered into a local inaccessible conformation. Finally, H30 shows low accessibility in the absence of factors but is very sensitive to perturbations such as upon addition of secondary proteins (Fig. 3G), ASOs (Fig. 4B, 2H) or RNA mutations (Fig. S13B). This suggests that the site does not have an intrinsic

propensity to form local RNA structure and, thus, its accessibility can be easily modulated (increased) by global perturbation of RNA structure.

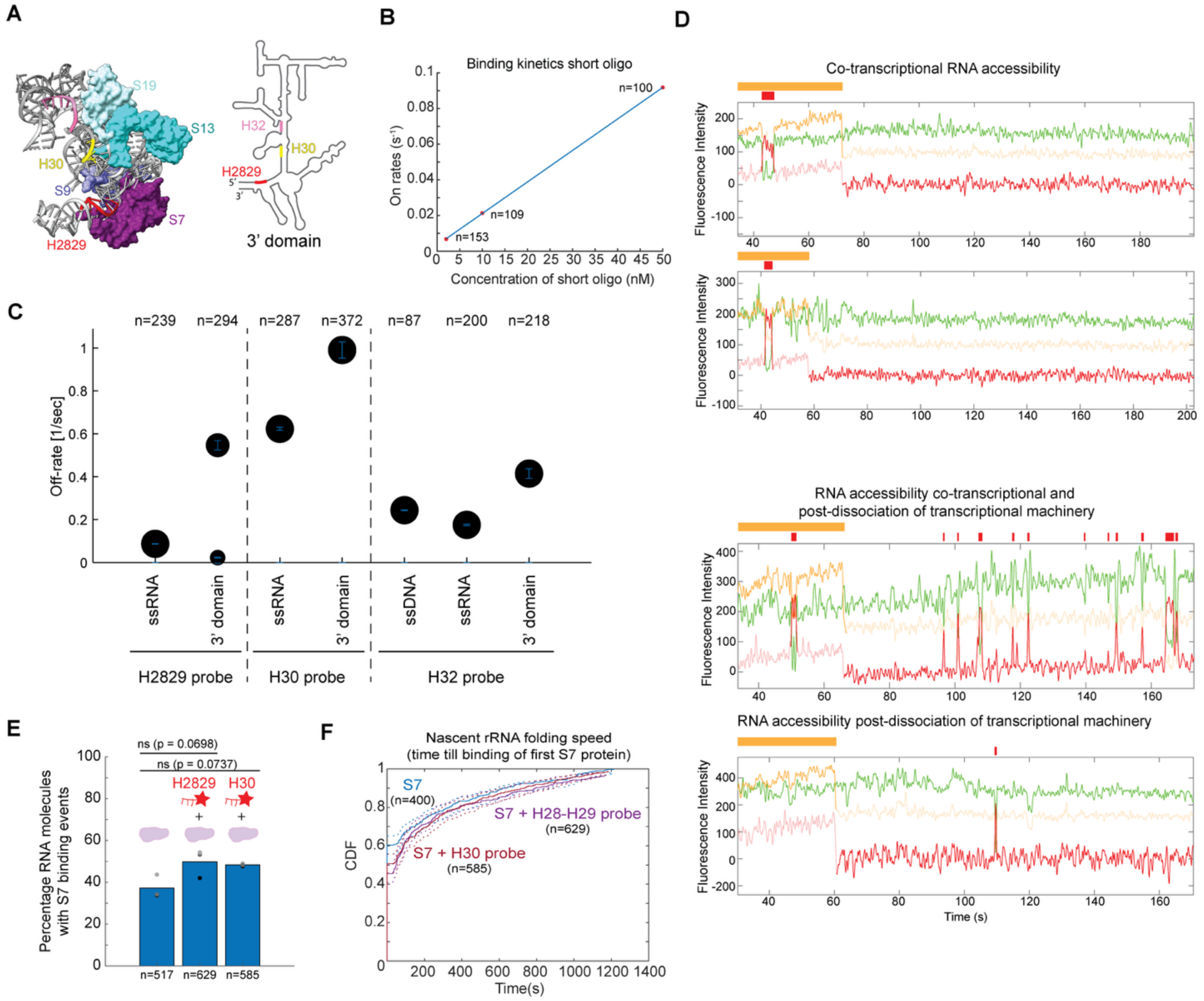

Fig. S1: Characterization of probe binding

(A) 3-dimensional structure (left) and secondary structure (right) of *E. coli* 30S ribosomal subunit zoomed into the S7 binding site. The regions probed in this study are shown: H2829 (red), H30 (yellow), H32 (pink) and surfaces of r-proteins S7 (purple), S9 (blue), S19 (sea blue) and S13 (cyan) (PDB accession code: 4V9P). **(B)** On-rate of the short DNA probe binding to H2829 ssRNA. **(C)** Comparison of the off-rate of the probes to the ssRNA, 3'domain and ssDNA. **(D)** Example smoothed traces showing binding of the H30 probe co-transcriptionally and post dissociation of the transcription machinery. Simplified representation of H30 probe binding events (red), and transcription signal (yellow) are shown above the trace. **(E)** The percentage of RNA molecules binding S7 (purple) in presence of the different short DNA probes (bars represent means of replicates). P-values (p) from two-sample Student's t-test (unequal variances): ns represents non-significant. **(F)** The cumulative distribution of the time from transcription start till appearance of the first S7 binding event in absence and presence of different short DNA probes (each curve is plotted by pooling data from 2 or 3 replicates, the dotted lines represent confidence bounds of the CDF fit). **(B,C,E,F)** Number of molecules analyzed (n) are shown.

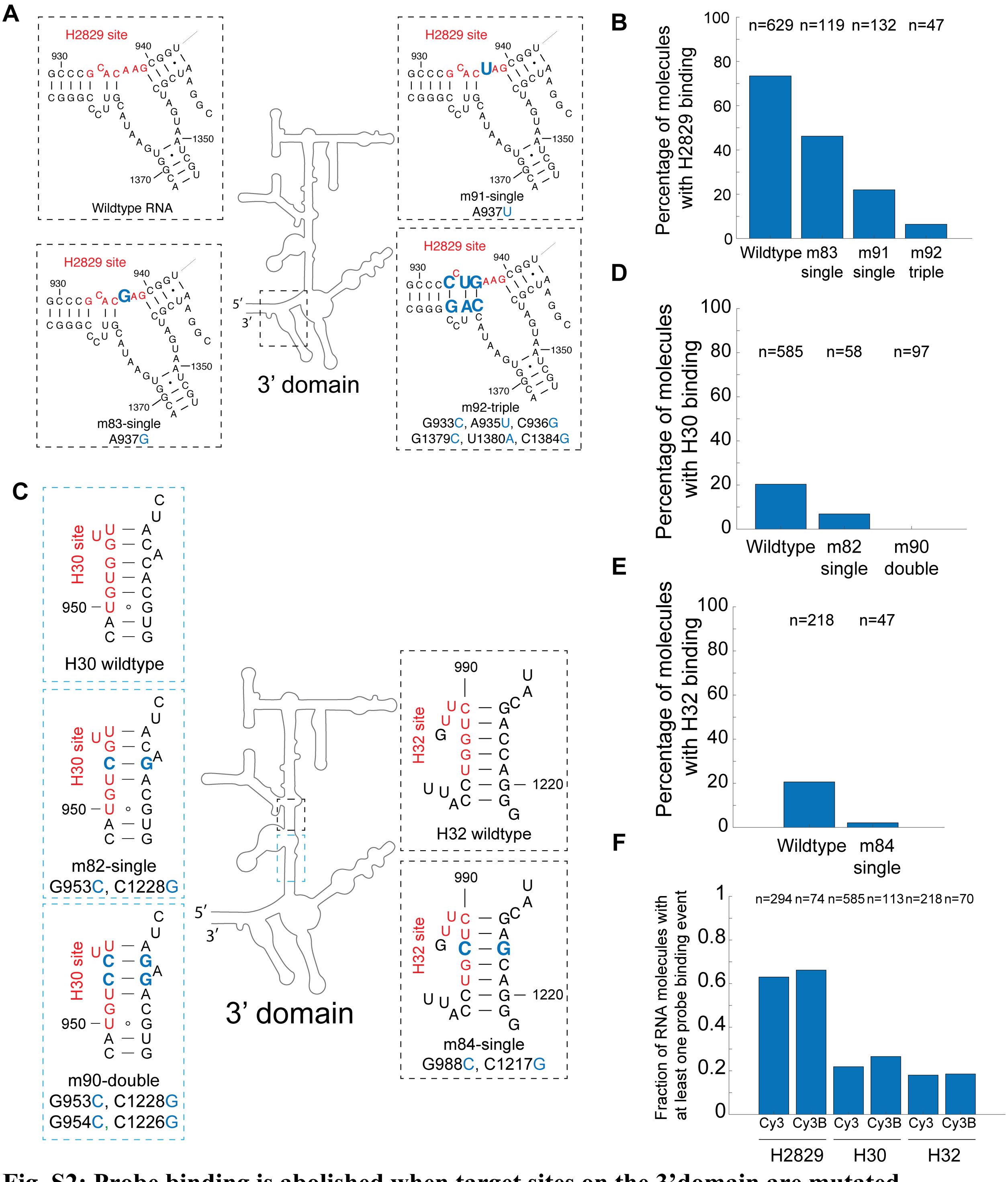

Fig. S2: Probe binding is abolished when target sites on the 3’domain are mutated. Schematic of 3’domain indicating the single, double or triple compensatory point mutations introduced (blue) and the target sites (red) for **(A)** H2829, **(C)** H30 and H32 target sites. Percentage of molecules accessible at **(B)** H2829, **(D)** H30, and **(E)** H32 sites to different constructs. **(F)** Comparison of the fraction of molecules with probe binding events to the 3’domain with Cy3 or Cy3B dye as donor and Cy5 dye as acceptor. Number of molecules analyzed (n) are shown. The data for wildtype (in **B,D,E**) and Cy3 (in **F**) is same as presented in Fig. 2B.

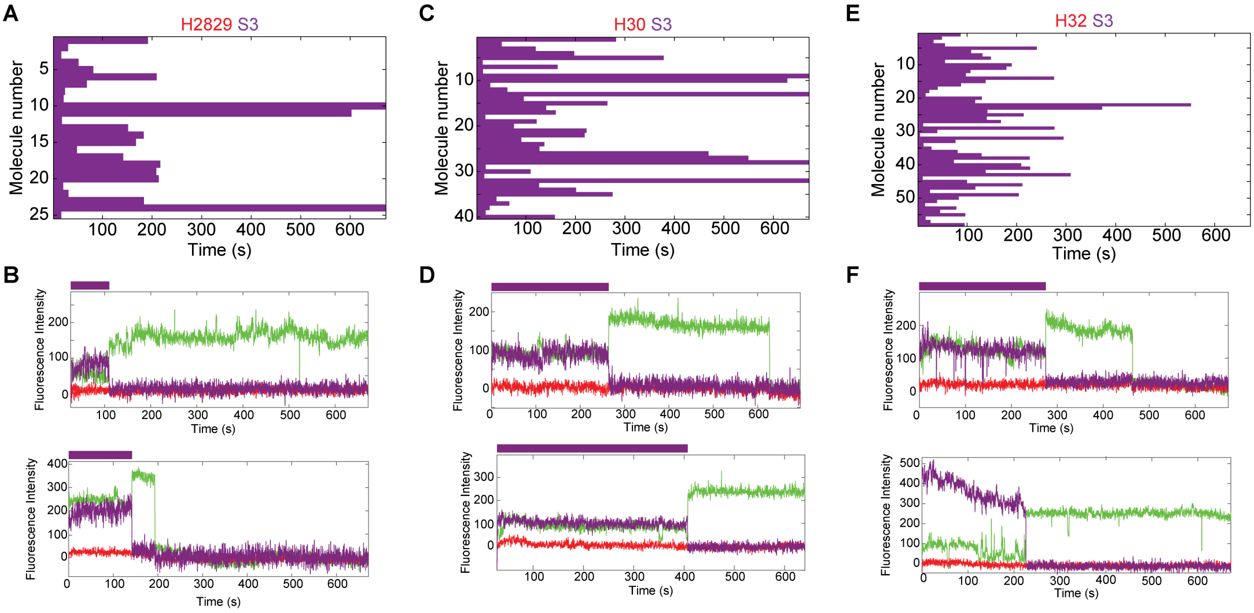

Fig. S3: RNA is inaccessible to probes in fully assembled 3'domain complex.
Probing RNA accessibility of the assembled 3'domain complex at **(A)** H2829, **(C)** H30 and **(E)** H32 sites, respectively. Example smoothed traces show bound S3 (purple – Cy5.5 dye), which is a marker for the fully assembled 3'domain (*25*). We did not detect probe binding (expected in red – Cy5 dye) to the subset of molecules with bound S3 for **(B)** H2829, **(D)** H30 and **(F)** H32 sites, respectively.

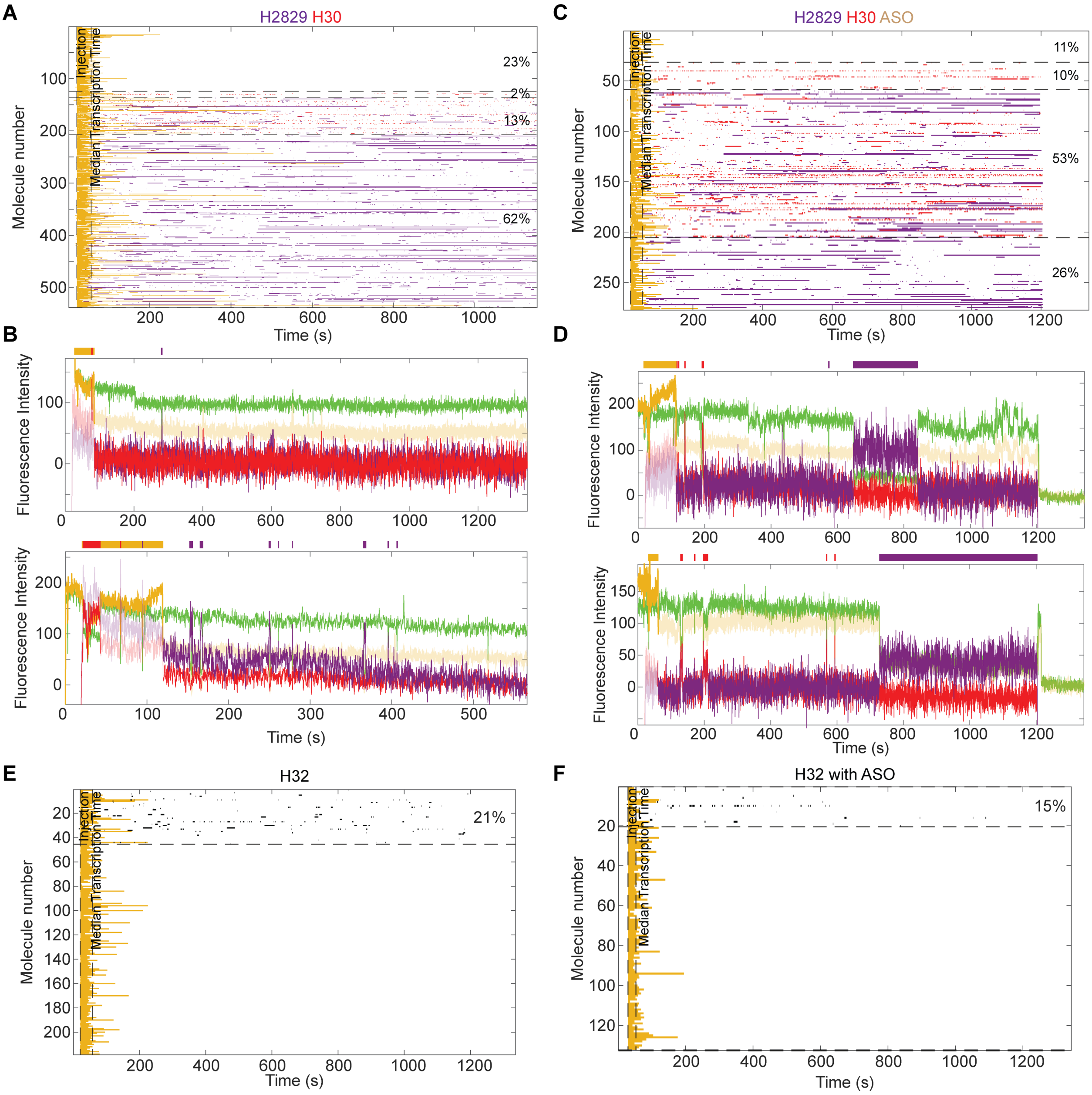

Fig. S4: Effect of ASOs on the RNA accessibility for different regions of the 3'domain rRNA. (A,C) Rasterplot showing individual molecules as rows and **(B,D)** smoothed example traces with transcription signal (yellow – Cy3.5 dye), H30 DNA probe (red – Cy5 dye) and H2829 DNA probe (purple – Cy5.5 dye) binding events shown as colored bars in absence **(A)** and presence of ASOs **(C)**, respectively**. (E)** Rasterplots showing individual molecules as rows with black representing H32 probe binding in absence **(E)** and presence of ASOs **(F)**. **(A,C,E)** plotted by pooling 2 replicates, **(F)** one experiment**.**

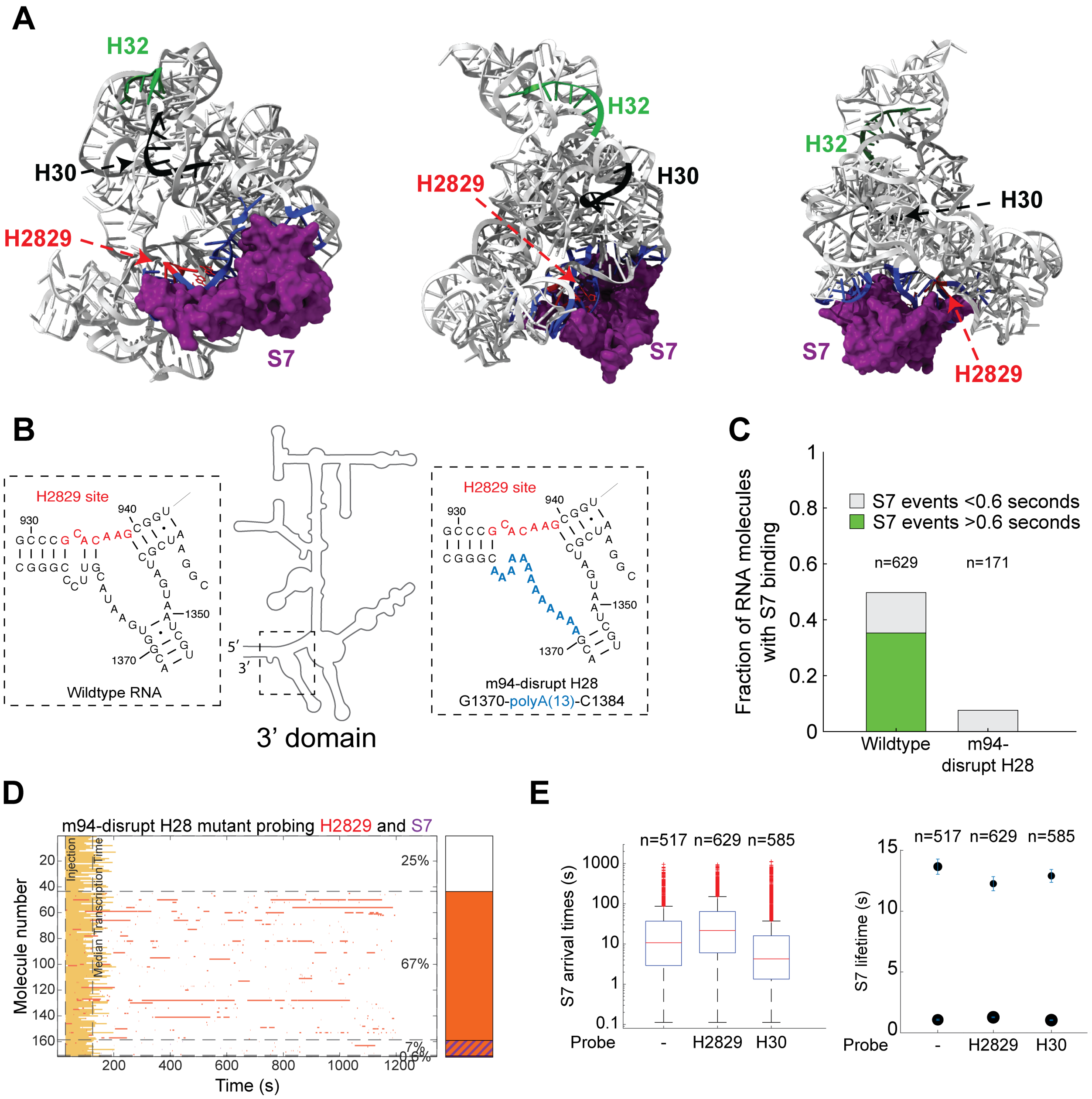

Fig. S5: RNA mutations strongly reduce S7 binding

(A) 3-dimensional structure of 3'domain bound by S7 from different angles. The regions probed in this study are shown: H2829 (red), H30 (black), H32 (green) and surfaces of r-proteins S7 (purple), contact sites of S7 to the RNA (blue) (PDB accession code: 4V9P). **(B)** Schematic of mutations on the RNA showing probe binding site (red) and mutations (blue). **(C)** Fraction of RNA molecules with S7 binding to wildtype and mutant RNA. Wildtype data is the same as presented in fig. S1E. **(D)** Rasterplot of m94-H28 disrupting mutant showing transcription (yellow – Cy3.5 dye), H2829 probe binding (red – Cy5 dye) and almost no S7 binding (purple – Cy5.5 dye). **(E)** S7 arrival times (left) and lifetimes (right) at 20 nM concentration of S7-Cy5.5 to the wildtype 3'domain RNA. Number of molecules analyzed (n) are shown.

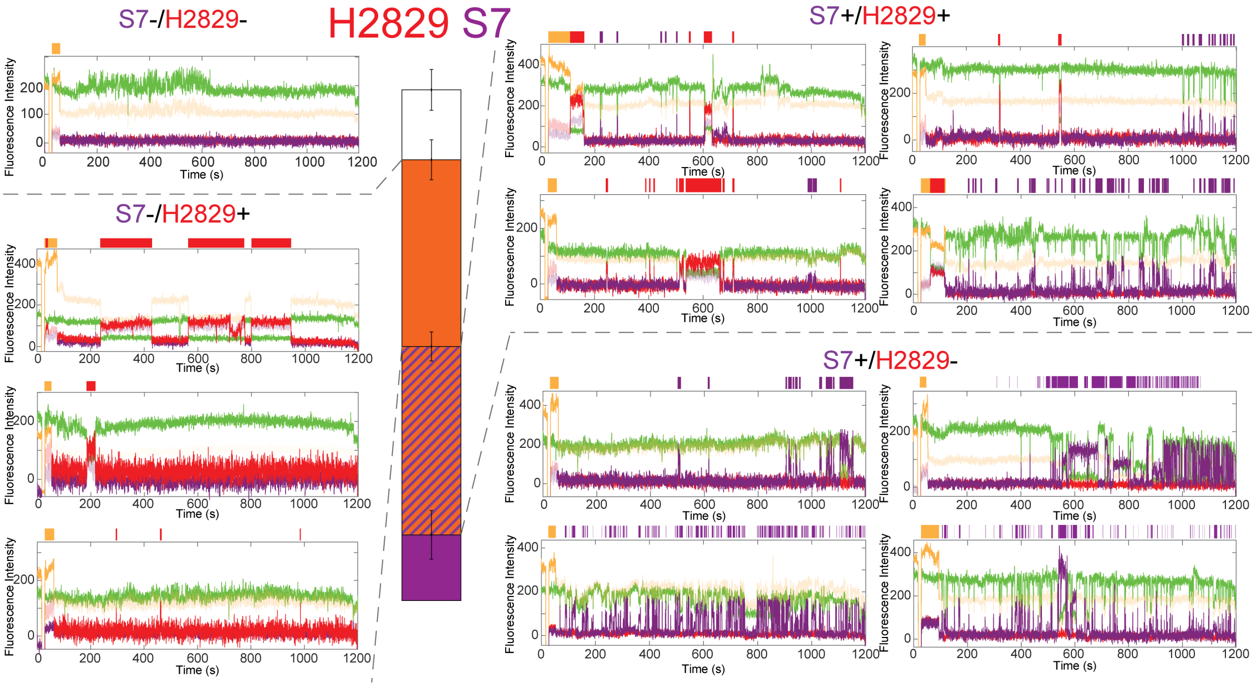

Fig. S6: H2829 accessibility and S7 binding is highly heterogeneous.
Example smoothed traces across different nascent RNA folding classes showing transcription (yellow – Cy3.5 dye), H2829 accessibility (red – Cy5 dye) and S7 binding (purple – Cy5.5 dye).

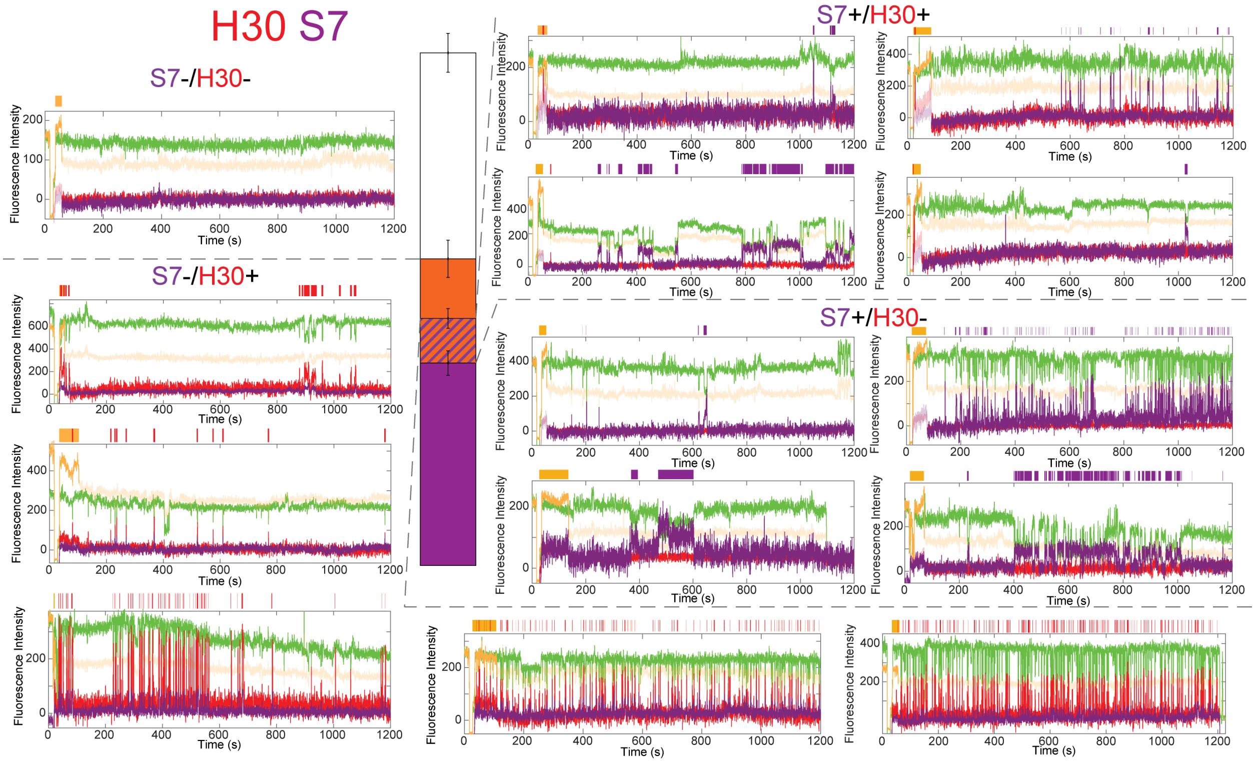

Fig. S7: H30 accessibility and S7 binding is highly heterogeneous.
Example smoothed traces across different nascent RNA folding classes showing transcription (yellow – Cy3.5 dye), H30 accessibility (red – Cy5 dye) and S7 binding (purple – Cy5.5 dye).

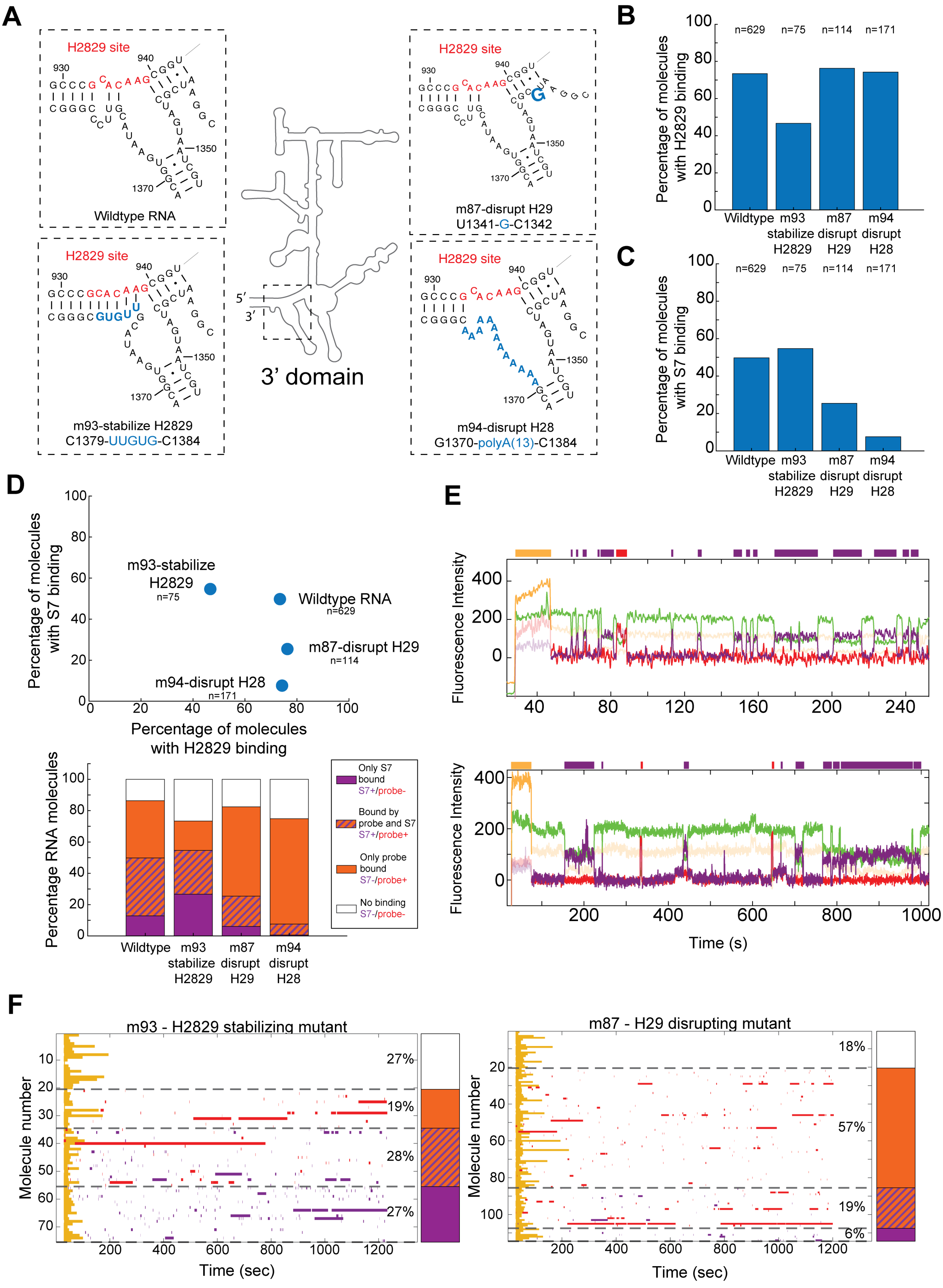

Fig. S8: Mutations on the H2829 region of the 3'domain shift the dynamic equilibrium. **(A)** Schematic of mutations on the RNA showing probe binding site (red) and mutations (blue). Percentage of molecules with **(B)** H2829 accessibility and **(C)** S7 binding in different constructs. Wildtype and m94 disrupting H28 mutant data in **(B,C,D)** is the same as presented

in Fig. 3G and fig. S5, respectively. **(D)** A scatter plot (top) represents the relative changes and, a stacked bar plot (bottom) represents the changes across classes observed in percentage molecules accessible at H2829 accessibility and S7 bound across different constructs. Number of molecules analyzed (n) in **(B-D)** are shown. **(E)** Example smoothed traces showing transcription (yellow – Cy3.5 dye) and dynamic equilibrium between H2829 being accessible (red – Cy5 dye) and S7 bound (purple – Cy5.5 dye). (**F**) Rasterplots showing the individual molecules as rows with transcription (yellow), H2829 probe (red) and S7 (purple) binding events for m93 (left) and m87 (right) mutants.

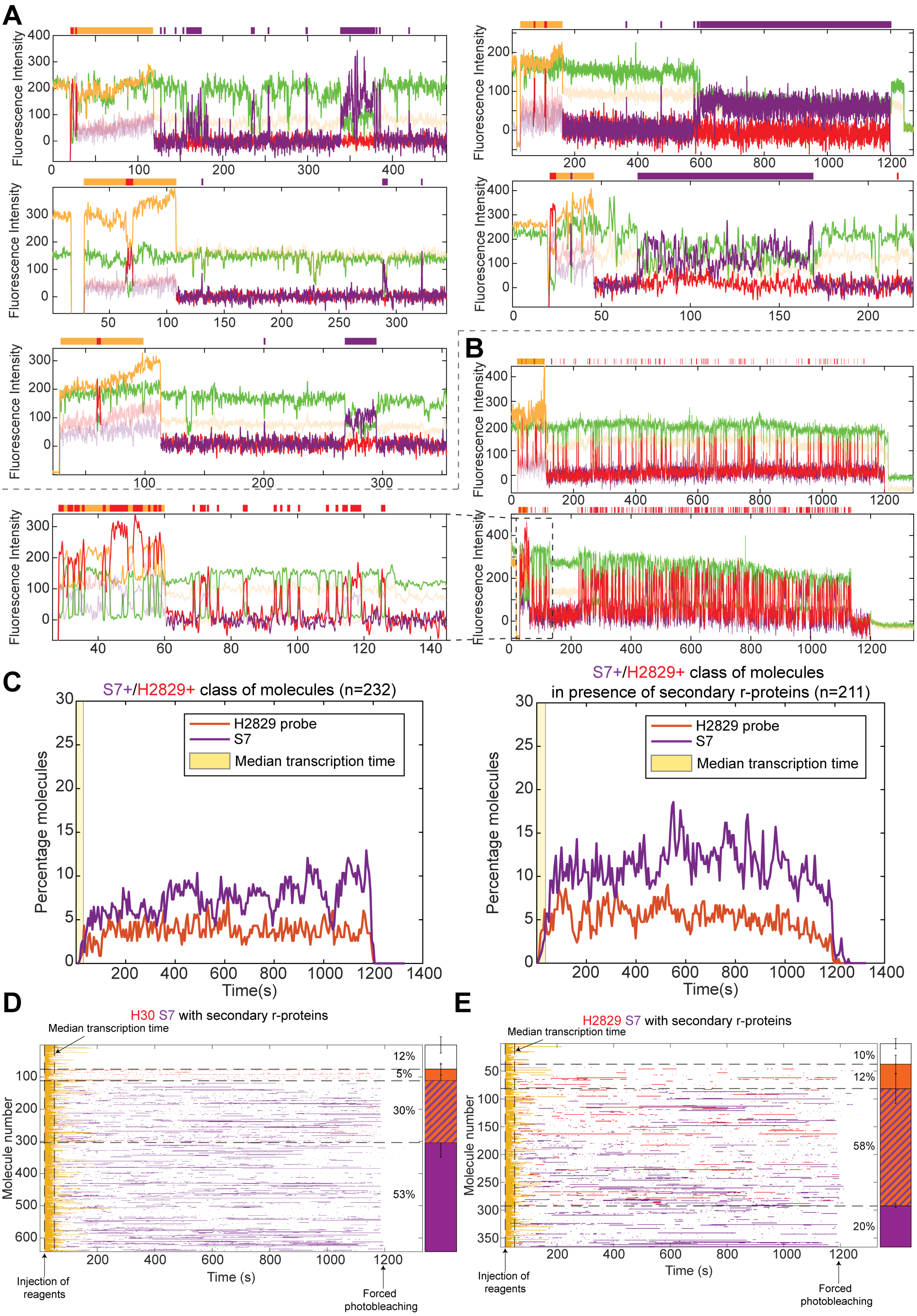

Fig. S9: Secondary r-proteins have variable effect on the different classes and on different regions of the RNA. Example smoothened traces of class **(A)** S7+/H30+ and **(B)** S7-/H30+ showing transcription (yellow – Cy3.5 dye), H30 probe binding (red – Cy5 dye) and S7 binding

(purple – Cy5.5 dye) in the presence of secondary r-proteins. **(C)** Time dependent analysis of the S7+/H2829+ class of molecules in absence (left) and presence of the secondary r-proteins (right). The region highlighted in yellow represents the median time RNA is associated to transcription machinery. Number of molecules analyzed (n) are shown. **(D,E)** Rasterplots showing the individual molecules as rows with transcription (yellow), DNA probe (red) and S7 (purple) binding events in presence of secondary r-proteins, shown as colored bars for H30 **(D)** and H2829 **(E)** sites, respectively. All data plotted by pooling 3 replicates. Error bars show weighted standard deviations.

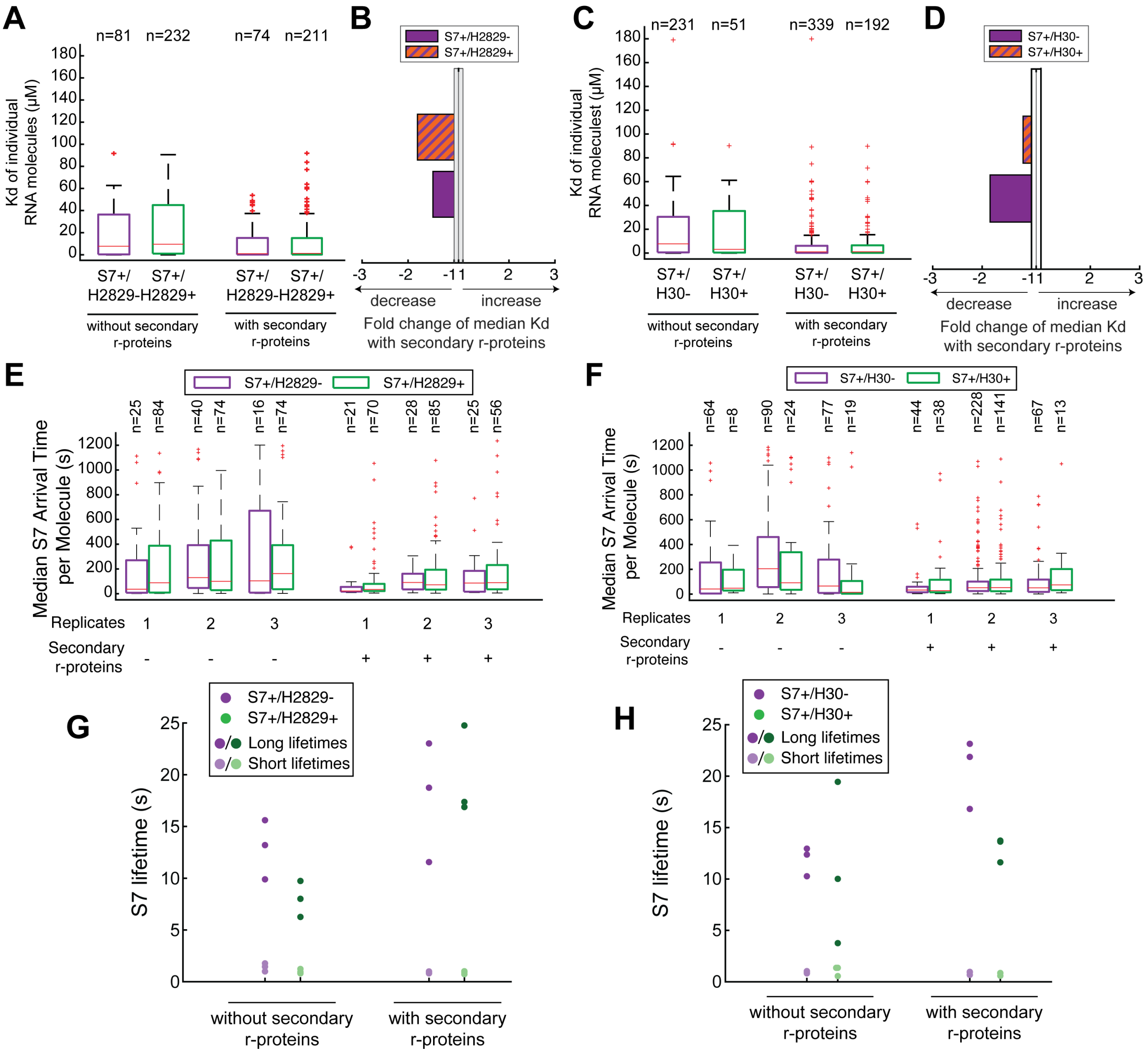

Fig. S10: Thermodynamics and kinetics of S7 across RNA folding classes.
(A,C) Boxplot of K_d for S7 of individual molecules calculated between 200 s and photobleaching and **(B,D)** fold change across nascent RNA folding classes in the absence and presence of secondary r-proteins for **(A,B)** H2829 site and **(C,D)** H30 site. **(E,F)** Median arrival time per molecule and **(G,H)** fits of S7-bound dwells to two exponential function across nascent RNA folding classes in the absence and presence of secondary r-proteins for **(E,G)** H2829 site and **(F,H)** H30 site. Different dots of same color represent replicates and shades of color represent different populations of S7 lifetimes in **(G,H)**. Number of molecules analyzed (n) are shown.

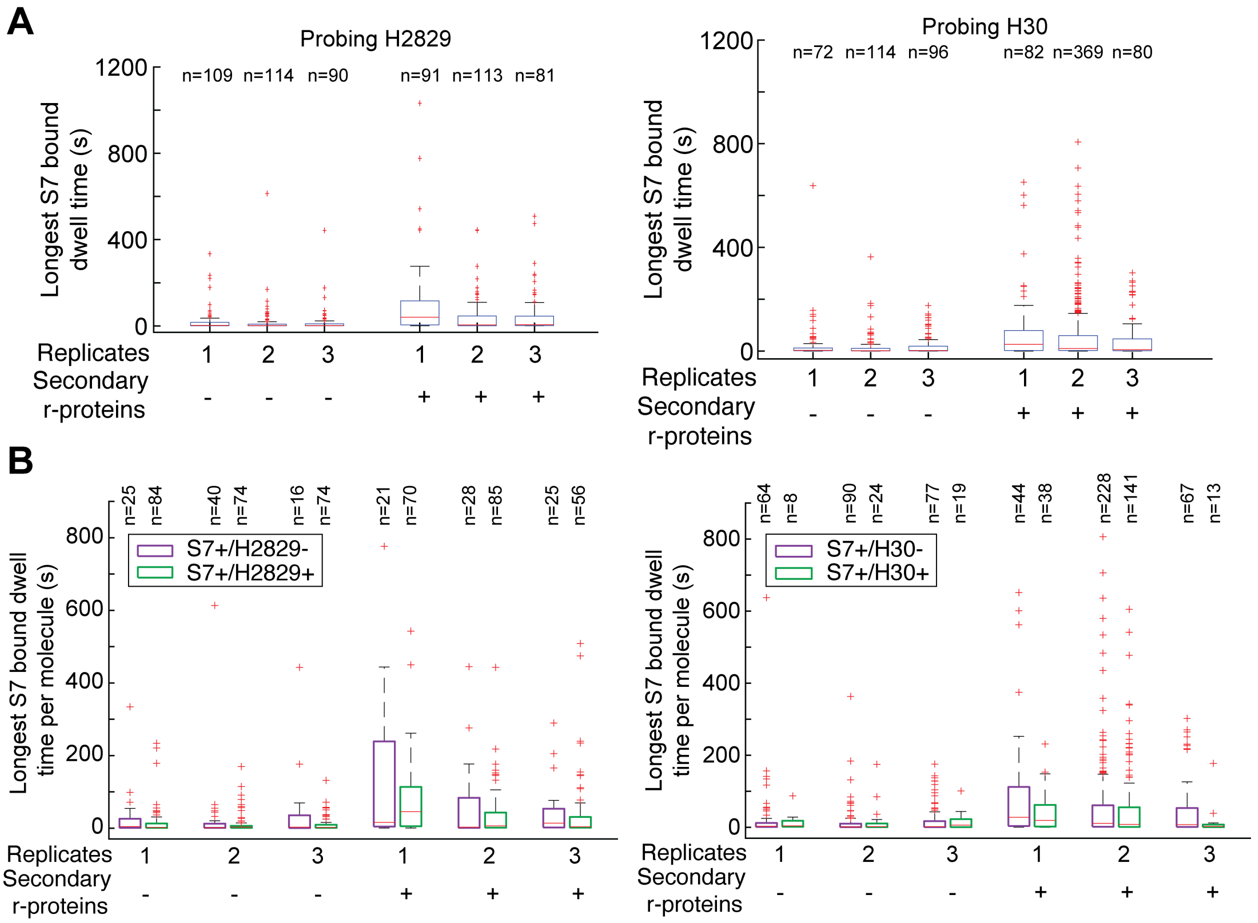

Fig. S11: Secondary r-proteins increase the longest S7 bound lifetime across all classes. Boxplot of **(A)** the longest S7 binding events of all the molecules, and **(B)** of molecules in individual classes for experiment probing H2829 (left) and H30 (right). Number of molecules analyzed (n) are shown. Plotting the longest S7 event per trace, instead of fitting all S7-bound dwell times (like in fig. S10), allows enrichment of events in which the complexes are assembled with secondary r-proteins. See more details in (*25*).

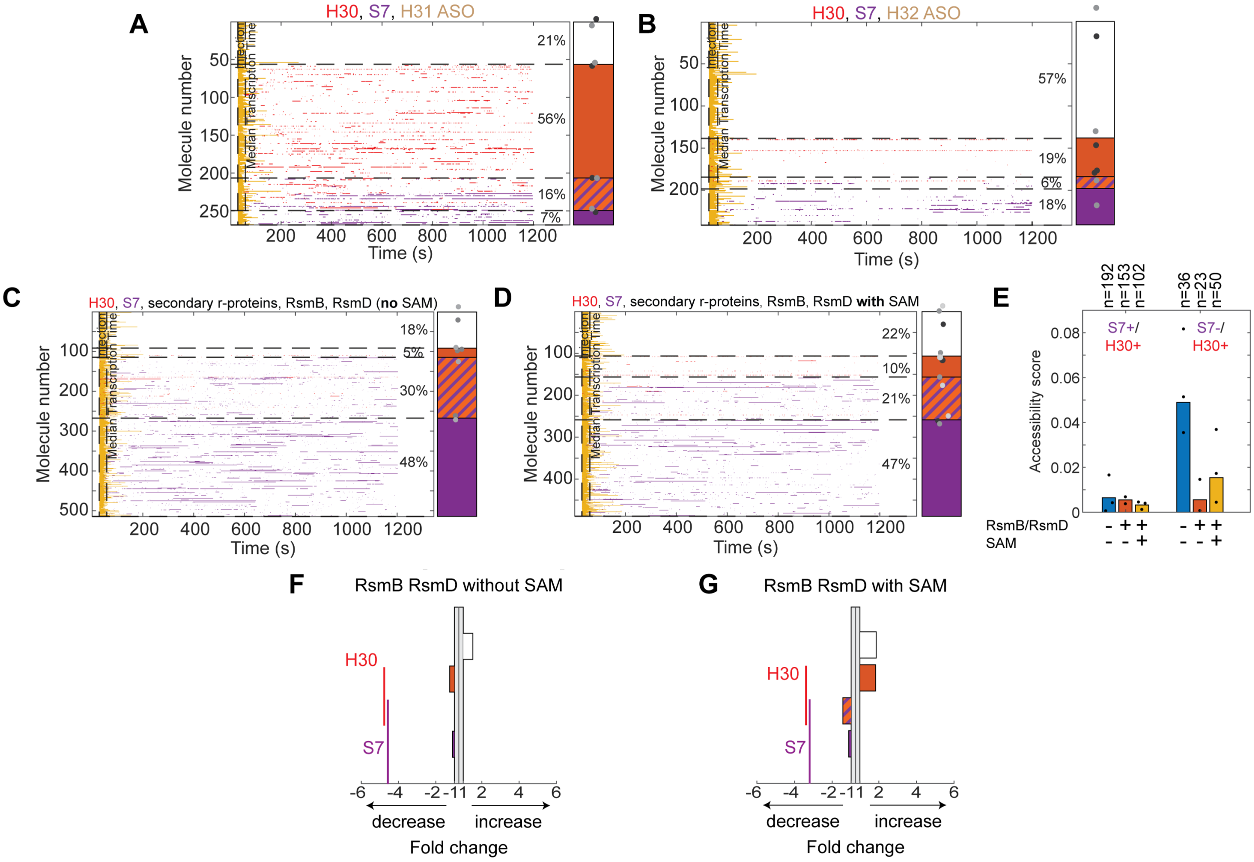

Fig. S12: Quantifying the modulation effect by ASOs and rRNA modification enzymes **(A-D)** Rasterplots showing individual molecules as rows for probing H30 region (red) and S7 binding (purple) on the 3'domain in presence of the H31 ASO **(A)** or H32 ASO **(B)** (both in absence of secondary r-proteins); or RsmB, RsmD without SAM **(C)** or with SAM **(D)** (both in presence of secondary r-proteins). Stacked bars are weighted means of replicates shown as shades of grey dots. **(E)** H30 accessibility score in RNA folding classes in presence of only secondary r-proteins (blue), in addition with RsmB and RsmD (orange) and in addition in presence of SAM (yellow). Mean accessibility score calculated by pooling 2 or 3 replicates. Dots represent individual replicates, number of molecules analyzed (n) are shown. **(F,G)** Relative fold-change in RNA class distributions upon addition of RsmB/RsmD in absence **(F)** or presence **(G)** of SAM (always in presence of S9, S13 and S19); data from Fig. 4E.

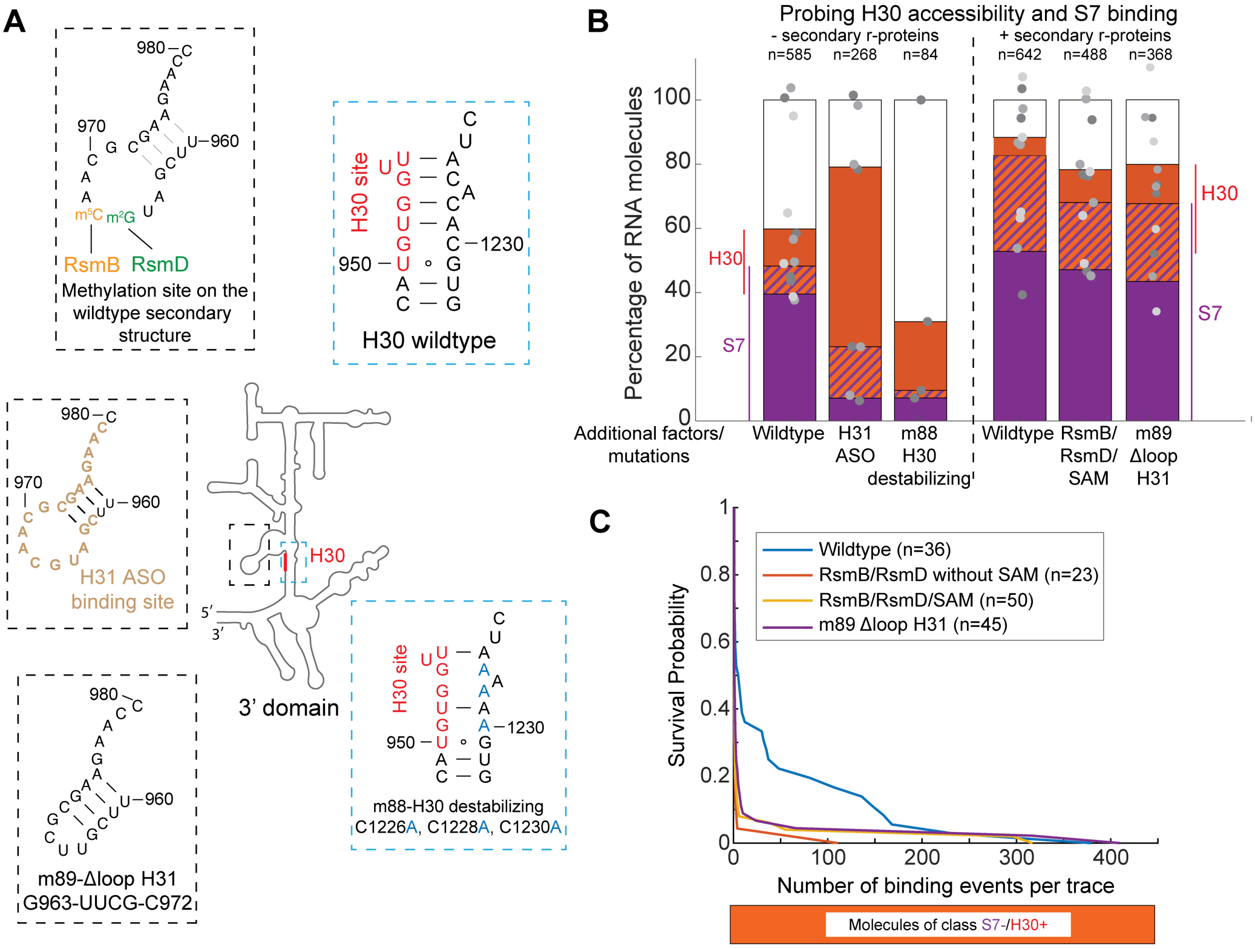

Fig. S13: H30 region is sensitive to mutations and RNA modification enzyme binding
(A) Schematic of mutations and ligand binding sites on the RNA. **(B)** Percentage of molecules in RNA folding classes affected by various perturbations. **(C)** Comparison of survival probability of the number of H30 binding events per trace in the S7-/H30+ class in the presence of secondary r-proteins. Dots represent replicates in **(B)** and n represents number of molecules. Data for wildtype with and without secondary r-proteins, and H31 ASO, RsmB/RsmD without and with SAM conditions is the same as presented in Fig. 3G and Fig. 4B,E, respectively.

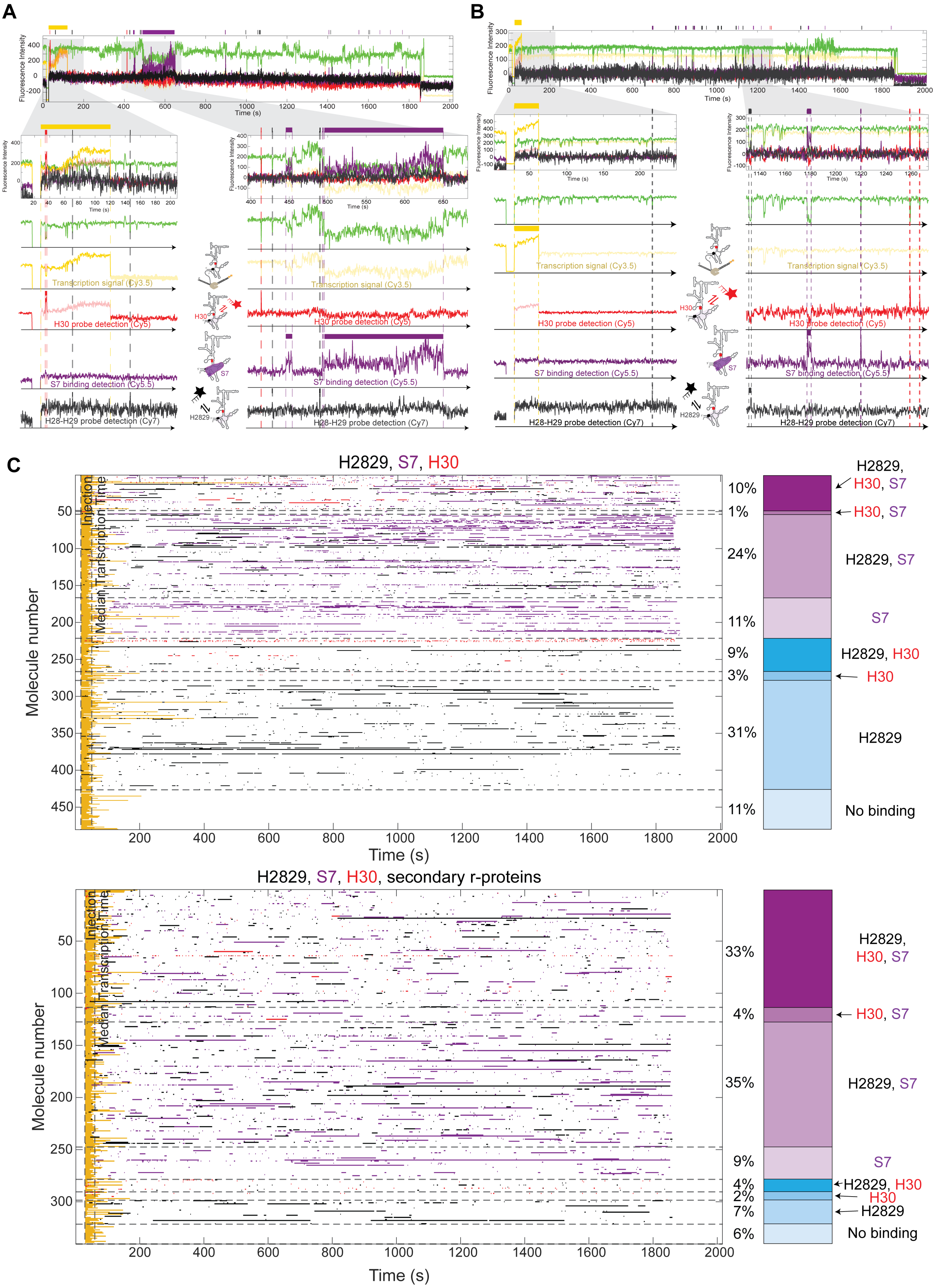

Fig. S14: Multi-site probing experiments show heterogeneity in RNA folding
(A,B) Example smoothed traces of the 5-color data showing FRET donor (green – Cy3 dye), transcription signal (yellow – Cy3.5 dye), H30 probe (red – Cy5 dye), S7 r-protein (purple –

Cy5.5 dye) and H2829 probe binding (black – Cy7 dye). Simplified representation of binding events is shown on top of the traces as bars. **(C)** Rasterplots of the multi-site probing experiments where individual molecules are rows showing transcription (yellow), binding of DNA probes to H30 (red) and H2829 (black), and r-protein S7 (purple) in absence (top) and presence of secondary r-proteins (bottom). Plotted by pooling 4 replicates – without, 2 replicates – with secondary r-proteins.

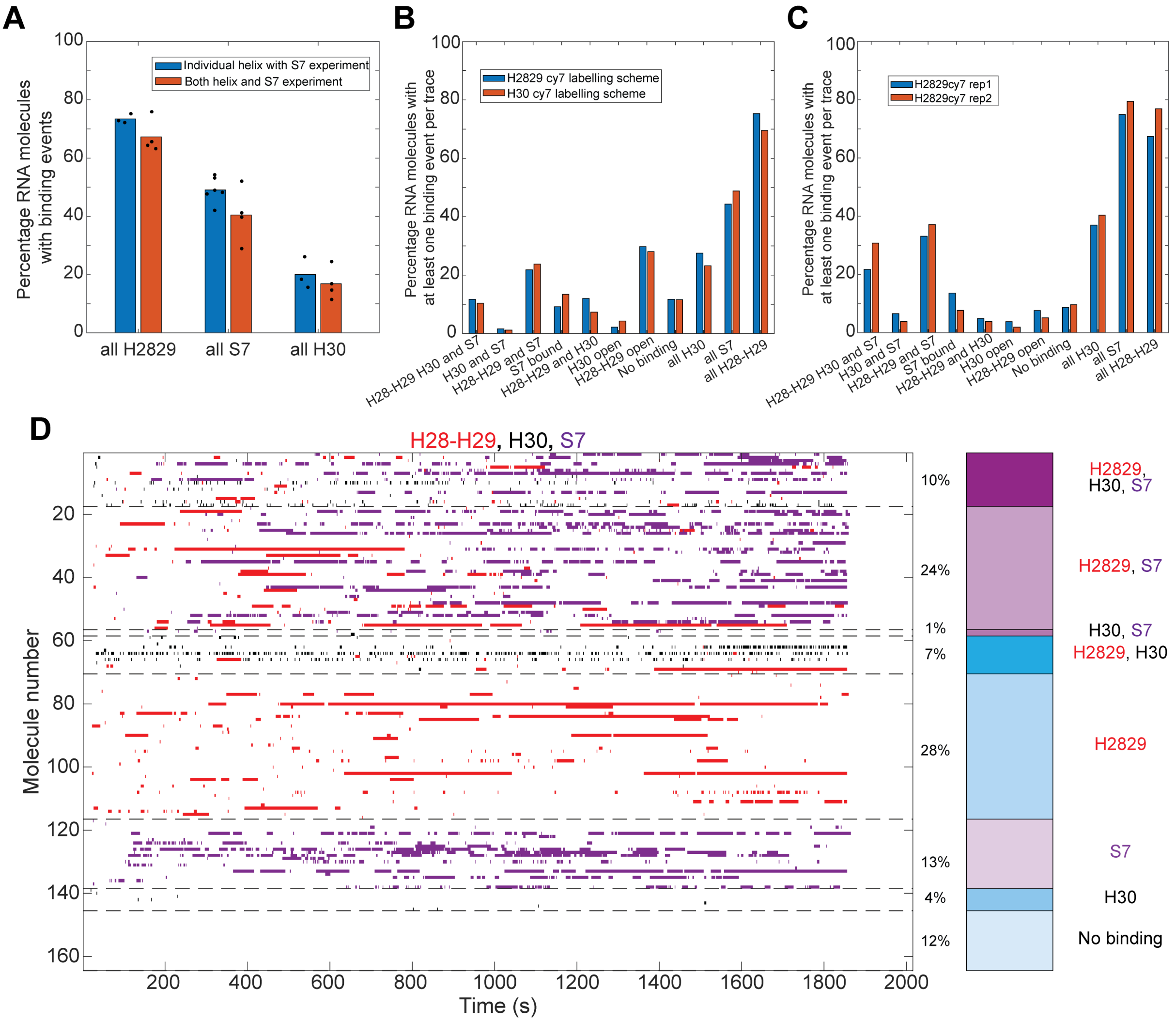

Fig. S15: Comparing multi-site probing experiments with single-site probing experiments **(A)** Percentage of molecules that show probe or S7 binding events in individual or two-site probing experiment in absence of secondary r-proteins. Bars represent mean of replicates shown as dots. **(B)** Comparing replicates using different dye labelling schemes in absence of secondary r-proteins (blue bars: Cy7-H2829-DNA probe, Cy5-H30-DNA probe and Cy5.5-S7; red bars: Cy5-H2829-DNA probe, Cy7-H30-DNA probe and Cy5.5-S7). **(C)** Comparing replicates for two-site probing with S7 binding in presence of secondary r-proteins (replicates are colored in blue and red). **(D)** Rasterplot of multi-site experiment where individual molecules are rows showing binding of DNA probe to H2829 (red), H30 (black) and S7 binding (purple) to the 3'domain in absence of secondary r-proteins; H2829-Cy5 dye, H30-Cy7 dye, S7-Cy5.5 dye labeling scheme.

Dataset	Koff,1	Koff,2	Percentage events described by koff,1
Duss et. al, Cell, 2019	0.79 ± 0.03 s-1	0.046 ± 0.004 s-1	75%
S7	0.94 ± 0.0045 s-1	0.073 ± 0.0035 s-1	63%
S7 + H2829 probe	0.79 ± 0.025 s-1	0.082 ± 0.0035 s-1	76%
S7 + H30 probe	0.97 ± 0.025 s-1	0.077 ± 0.0035 s-1	76%

Table S1: Comparison of S7 off-rates from this study to previous study.

Materials

Overview of artificial DNA sequences used.

Backbone of the DNA template used.
RNAP promoter (green), hybridization sequence for immobilization of stalled complex through 5'-end of nascent RNA (red), binding site for DNA oligo binding to 5'end of nascent RNA (blue), 3'domain sequence (see further below), binding site for labeled DNA oligo binding to 3'-end of nascent RNA (orange) and triple transcription terminator (black).

The DNA sequence is:
ctggcagttt taggctgatt tggttgaatg ttgcgcggtc agaaaattat tttaaatttc ctcttgtcag gccggaataa ctccctataa tgcgccacc ACT ACCAC CACCC AACCA ACACA CC AAC CAC TCC AAT TAC ATA CAC C (3'domain) CCCTA TCCCT TATCT TAAC GGCTCCTTTTGGAGCCTTTTTTTTTGGAGATTTTCTAAAACGAAAGGCTCAGT CGAAAGACTGGGCCTTTCGTTTTATCT TAATC ACACT GGCTC ACCTT CGGGT GGGCC TTTCT GCGTT TAT

3'domain (5' to 3'):
tgacgggggc ccgcacaagc ggtggagcat gtggtttaat tcgatgcaac gcgaagaacc ttacctggtc ttgacatcca cggaagtttt cagagatgag aatgtgcctt cgggaaccgt gagacaggtg ctgcatggct gtcgtcagct cgtgttgtga aatgttgggt taagtcccgc aacgagcgca acccttatcc tttgttgcca gcggtccggc cgggaactca aaggagactg ccagtgataa actggaggaa ggtggggatg acgtcaagtc atcatggccc ttacgaccag ggctacacac gtgctacaat ggcgcataca aagagaagcg acctcgcgag agcaagcgga cctcataaag tgcgtcgtag tccggattgg agtctgcaac tcgactccat gaagtcggaa tcgctagtaa tcgtggatca gaatgccacg gtgaatacgt tcccgggcct tgtaca

m82 (5' to 3'):
tgacgggggcccgcacaagcggtggagcatgtCgtttaattcgatgcaacgcgaagaaccttacctggtcttgacatccacggaagtt
ttcagagatgagaatgtgccttcgggaaccgtgagacaggtgctgcatggctgtcgtcagctcgtgttgtgaaatgttgggttaagtccc
gcaacgagcgcaacccttatcctttgttgccagcggtccggccgggaactcaaaggagactgccagtgataaactggaggaaggtg
gggatgacgtcaagtcatcatggcccttacgaccagggctacaGacgtgctacaatggcgcatacaaagagaagcgacctcgcga
gagcaagcggacctcataaagtgcgtcgtagtccggattggagtctgcaactcgactccatgaagtcggaatcgctagtaatcgtgga
tcagaatgccacggtgaatacgttcccgggccttgtaca

m83 (5' to 3'):
tgacgggggcccgcacGagcggtggagcatgtggtttaattcgatgcaacgcgaagaaccttacctggtcttgacatccacggaagt
tttcagagatgagaatgtgccttcgggaaccgtgagacaggtgctgcatggctgtcgtcagctcgtgttgtgaaatgttgggttaagtcc
cgcaacgagcgcaacccttatcctttgttgccagcggtccggccgggaactcaaaggagactgccagtgataaactggaggaaggt
ggggatgacgtcaagtcatcatggcccttacgaccagggctacacacgtgctacaatggcgcatacaaagagaagcgacctcgcga
gagcaagcggacctcataaagtgcgtcgtagtccggattggagtctgcaactcgactccatgaagtcggaatcgctagtaatcgtgga
tcagaatgccacggtgaatacgttcccgggccttgtaca

m84 (5' to 3'):
tgacgggggcccgcacaagcggtggagcatgtggtttaattcgatgcaacgcgaagaaccttacctgCtcttgacatccacggaagtt
ttcagagatgagaatgtgccttcgggaaccgtgagacaggtgctgcatggctgtcgtcagctcgtgttgtgaaatgttgggttaagtccc
gcaacgagcgcaacccttatcctttgttgccagcggtccggccgggaactcaaaggagactgccagtgataaactggaggaaggtg
gggatgacgtcaagtcatcatggcccttacgaGcagggctacacacgtgctacaatggcgcatacaaagagaagcgacctcgcga
gagcaagcggacctcataaagtgcgtcgtagtccggattggagtctgcaactcgactccatgaagtcggaatcgctagtaatcgtgga
tcagaatgccacggtgaatacgttcccgggccttgtaca

m87 (5' to 3'):
tgacgggggcccgcacaagcggtggagcatgtggtttaattcgatgcaacgcgaagaaccttacctggtcttgacatccacggaagtt
ttcagagatgagaatgtgccttcgggaaccgtgagacaggtgctgcatggctgtcgtcagctcgtgttgtgaaatgttgggttaagtccc
gcaacgagcgcaacccttatcctttgttgccagcggtccggccgggaactcaaaggagactgccagtgataaactggaggaaggtg
gggatgacgtcaagtcatcatggcccttacgaccagggctacacacgtgctacaatggcgcatacaaagagaagcgacctcgcgag
agcaagcggacctcataaagtgcgtcgtagtccggattggagtctgcaactcgactccatgaagtcggaatGcgctagtaatcgtgga
tcagaatgccacggtgaatacgttcccgggccttgtaca

m88 (5' to 3'):
tgacgggggcccgcacaagcggtggagcatgtggtttaattcgatgcaacgcgaagaaccttacctggtcttgacatccacggaagtt
ttcagagatgagaatgtgccttcgggaaccgtgagacaggtgctgcatggctgtcgtcagctcgtgttgtgaaatgttgggttaagtccc
gcaacgagcgcaacccttatcctttgttgccagcggtccggccgggaactcaaaggagactgccagtgataaactggaggaaggtg
gggatgacgtcaagtcatcatggcccttacgaccagggctaAaAaAgtgctacaatggcgcatacaaagagaagcgacctcgcg
agagcaagcggacctcataaagtgcgtcgtagtccggattggagtctgcaactcgactccatgaagtcggaatcgctagtaatcgtgg
atcagaatgccacggtgaatacgttcccgggccttgtaca

m89 (5' to 3'):
tgacgggggcccgcacaagcggtggagcatgtggtttaattcgTTcgcgaagaaccttacctggtcttgacatccacggaagttttca
gagatgagaatgtgccttcgggaaccgtgagacaggtgctgcatggctgtcgtcagctcgtgttgtgaaatgttgggttaagtcccgca
acgagcgcaacccttatcctttgttgccagcggtccggccgggaactcaaaggagactgccagtgataaactggaggaaggtgggg
atgacgtcaagtcatcatggcccttacgaccagggctacacacgtgctacaatggcgcatacaaagagaagcgacctcgcgagagc
aagcggacctcataaagtgcgtcgtagtccggattggagtctgcaactcgactccatgaagtcggaatcgctagtaatcgtggatcaga
atgccacggtgaatacgttcccgggccttgtaca

m90 (5' to 3'):
tgacgggggcccgcacaagcggtggagcatgtCCtttaattcgatgcaacgcgaagaaccttacctggtcttgacatccacggaagt
tttcagagatgagaatgtgccttcgggaaccgtgagacaggtgctgcatggctgtcgtcagctcgtgttgtgaaatgttgggttaagtcc
cgcaacgagcgcaacccttatcctttgttgccagcggtccggccgggaactcaaaggagactgccagtgataaactggaggaaggt
ggggatgacgtcaagtcatcatggcccttacgaccagggctaGaGacgtgctacaatggcgcatacaaagagaagcgacctcgcg
agagcaagcggacctcataaagtgcgtcgtagtccggattggagtctgcaactcgactccatgaagtcggaatcgctagtaatcgtgg
atcagaatgccacggtgaatacgttcccgggccttgtaca

m91 (5' to 3'):
tgacgggggcccgcacTagcggtggagcatgtggtttaattcgatgcaacgcgaagaaccttacctggtcttgacatccacggaagtt
ttcagagatgagaatgtgccttcgggaaccgtgagacaggtgctgcatggctgtcgtcagctcgtgttgtgaaatgttgggttaagtccc
gcaacgagcgcaacccttatcctttgttgccagcggtccggccgggaactcaaaggagactgccagtgataaactggaggaaggtg
gggatgacgtcaagtcatcatggcccttacgaccagggctacacacgtgctacaatggcgcatacaaagagaagcgacctcgcgag
agcaagcggacctcataaagtgcgtcgtagtccggattggagtctgcaactcgactccatgaagtcggaatcgctagtaatcgtggatc
agaatgccacggtgaatacgttcccgggccttgtaca

m92 (5' to 3'):
tgacgggggcccCcTGaagcggtggagcatgtggtttaattcgatgcaacgcgaagaaccttacctggtcttgacatccacggaag
ttttcagagatgagaatgtgccttcgggaaccgtgagacaggtgctgcatggctgtcgtcagctcgtgttgtgaaatgttgggttaagtcc
cgcaacgagcgcaacccttatcctttgttgccagcggtccggccgggaactcaaaggagactgccagtgataaactggaggaaggt
ggggatgacgtcaagtcatcatggcccttacgaccagggctacacacgtgctacaatggcgcatacaaagagaagcgacctcgcga
gagcaagcggacctcataaagtgcgtcgtagtccggattggagtctgcaactcgactccatgaagtcggaatcgctagtaatcgtgga
tcagaatgccacggtgaatacCAtccGgggccttgtaca

m93 (5' to 3'):

tgacgggggcccgcacaagcggtggagcatgtggtttaattcgatgcaacgcgaagaaccttacctggtcttgacatccacggaagtt
ttcagagatgagaatgtgccttcgggaaccgtgagacaggtgctgcatggctgtcgtcagctcgtgttgtgaaatgttgggttaagtccc
gcaacgagcgcaacccttatcctttgttgccagcggtccggccgggaactcaaaggagactgccagtgataaactggaggaaggtg
gggatgacgtcaagtcatcatggcccttacgaccagggctacacacgtgctacaatggcgcatacaaagagaagcgacctcgcgag
agcaagcggacctcataaagtgcgtcgtagtccggattggagtctgcaactcgactccatgaagtcggaatcgctagtaatcgtggatc
agaatgccacggtgaatacTTGTGcgggccttgtaca

m94 (5' to 3'):
tgacgggggcccgcacaagcggtggagcatgtggtttaattcgatgcaacgcgaagaaccttacctggtcttgacatccacggaagtt
ttcagagatgagaatgtgccttcgggaaccgtgagacaggtgctgcatggctgtcgtcagctcgtgttgtgaaatgttgggttaagtccc
gcaacgagcgcaacccttatcctttgttgccagcggtccggccgggaactcaaaggagactgccagtgataaactggaggaaggtg
gggatgacgtcaagtcatcatggcccttacgaccagggctacacacgtgctacaatggcgcatacaaagagaagcgacctcgcgag
agcaagcggacctcataaagtgcgtcgtagtccggattggagtctgcaactcgactccatgaagtcggaatcgctagtaatcgtggatc
agaatgccacgAAAAAAAAAAAAAcgggccttgtaca

Table S2: List of modified DNA oligos.

Oligonucleotide ID	DNA/RNA Sequence (5' to 3')
p0030_ab_fw	TCACGAAAGCTGAGTAGTCACGAGTCTTCT/idSp/CTGGCAGTTTTAGGCTGATTTGG
p0075_ab_bw	CCTTAATCATACTCACCAAATTACCATCCC/idSp/ATAAACGCAGAAAGGCCCAC
p0088_2xCy3.5	Cy3p5-GGGATGGTAATTTGG[dT-Cy3p5]GAGTATGATTAAGG
p0109-biotin	5BiotinTEG/TTATCCGCTCACAATTCCACA
p44	GGTGTGTTGGTTGGGTGGTGGTAGT/idSp/GCAGGTCGACTCTAGAGGAT
p66-Cy3-FQ	/5Cy3/GGTGTATGTAATTGGAGTGGTT/3IABkFQ/
P153-Cy3B-FQ	/5Cy3B/GGTGTATGTAATTGGAGTGGTT/3IABkFQ/
P112A	5BiotinTEG/AAAAAAcgcacaagAAA/3Cy3sp/
prKG047	/5Cy3/AAAuguggguuuAAAAAA/3biotinTEG/
prKG078	5BiotinTEG/AAAAAAccuggucuAAA/3Cy3sp/
prKG081	5BiotinTEG/AAAAAAcctggtctAAA/3Cy3sp/
p110Bcy5	CTTGTGC/3Cy5sp/
prKG058cy5.5	CTTGTGC/3Cy55sp/
prKG080cy7	CTTGTGC/3Cy7p/
prKG032cy5	/5Cy5/AACCACA
prKG057cy7	/5Cy7/AACCACA
prKG053cy5	AGACCAG/3Cy5sp/
prKG072	TGACGACAGCCATGCAGC

prKG073	GTAGCCCTGGTCGTAAGG
prKG074	GTTCTTCGCGTTGCATCG
prKG075	TGTATGCGCCATTGTAGC
prKG076	CGATTACTAGCGATTCCG
prKG077	TGTACAAGGCCCGGGAAC

/idSp/ denotes abasic site.
[dT-Cy3p5] is an internal modification labeled on the nucleobase of deoxythymidine.
5BiotinTEG denotes Biotin-TEG attached to 5'-end
3BiotinTEG denotes Biotin-TEG attached to 3'-end
/3IABkFQ/ denotes FQ quencher attached to 3'-end
/5Cy3/ denotes Cy3 label attached to 5'-end
/5Cy3B/ denotes Cy3B label attached to 5'-end
/3Cy3sp/ denotes Cy3 label attached to 3'-end
/5Cy5/ denotes Cy5 label attached to 5'-end
/3Cy5sp/ denotes Cy5 label attached to 3'-end
/3Cy55Sp/ denotes Cy5.5 label attached to 3'-end
/3Cy7p/ denotes Cy7 label attached to 3'-end
/5Cy7/ denotes Cy7 label attached to 5'-end

REFERENCES

1. D. E. Draper, A guide to ions and RNA structure. *RNA* **10**, 335–343 (2004).

2. T. Ha, X. Zhuang, H. D. Kim, J. W. Orr, J. R. Williamson, S. Chu, Ligand-induced conformational changes observed in single RNA molecules. *Proc. Natl. Acad. Sci. U.S.A.* **96**, 9077–9082 (1999).

3. M. M. Balas, E. W. Hartwick, C. Barrington, J. T. Roberts, S. K. Wu, R. Bettcher, A. M. Griffin, J. S. Kieft, A. M. Johnson, Establishing RNA-RNA interactions remodels lncRNA structure and promotes PRC2 activity. *Sci. Adv.* **7**, eabc9191 (2021).

4. Y. Lou, S. A. Woodson, Co-transcriptional folding of the glmS ribozyme enables a rapid response to metabolite. *Nucleic Acids Res.* **52**, 872–884 (2024).

5. J. Ding, Y.-T. Lee, Y. Bhandari, C. D. Schwieters, L. Fan, P. Yu, S. G. Tarosov, J. R. Stagno, B. Ma, R. Nussinov, A. Rein, J. Zhang, Y.-X. Wang, Visualizing RNA conformational and architectural heterogeneity in solution. *Nat. Commun.* **14**, 714 (2023).

6. Q. Zhang, A. C. Stelzer, C. K. Fisher, H. M. Al-Hashimi, Visualizing spatially correlated dynamics that directs RNA conformational transitions. *Nature* **450**, 1263–1267 (2007).

7. J. Abramson, J. Adler, J. Dunger, R. Evans, T. Green, A. Pritzel, O. Ronneberger, L. Willmore, A. J. Ballard, J. Bambrick, S. W. Bodenstein, D. A. Evans, C.-C. Hung, M. O'Neill, D. Reiman, K. Tunyasuvunakool, Z. Wu, A. Žemgulytė, E. Arvaniti, C. Beattie, O. Bertolli, A. Bridgland, A. Cherepanov, M. Congreve, A. I. Cowen-Rivers, A. Cowie, M. Figurnov, F. B. Fuchs, H. Gladman, R. Jain, Y. A. Khan, C. M. R. Low, K. Perlin, A. Potapenko, P. Savy, S. Singh, A. Stecula, A. Thillaisundaram, C. Tong, S. Yakneen, E. D. Zhong, M. Zielinski, Augustin Žídek, V. Bapst, P. Kohli, M. Jaderberg, D. Hassabis, J. M. Jumper, Accurate structure prediction of biomolecular interactions with AlphaFold 3. *Nature* **630**, 493–500 (2024).

8. J. Jumper, R. Evans, A. Pritzel, T. Green, M. Figurnov, O. Ronneberger, K. Tunyasuvunakool, R. Bates, A. Zidek, A. Potapenko, A. Bridgland, C. Meyer, S. A. A. Kohl, A. J. Ballard, A. Cowie, B. Romera-Paredes, S. Nikolov, R. Jain, J. Adler, T. Back, S. Petersen, D. Reiman, E. Clancy,

M. Zielinski, M. Steinegger, M. Pacholska, T. Berghammer, S. Bodenstein, D. Silver, O. Vinyals, A. W. Senior, K. Kavukcuoglu, P. Kohli, D. Hassabis, Highly accurate protein structure prediction with AlphaFold. *Nature* **596**, 583–589 (2021).

9. R. J. L. Townshend, S. Eismann, A. M. Watkins, R. Rangan, M. Karelina, R. Das, R. O. Dror, Geometric deep learning of RNA structure. *Science* **373**, 1047–1051 (2021).

10. T. Shen, Z. Hu, S. Sun, D. Liu, F. Wong, J. Wang, J. Chen, Y. Wang, L. Hong, J. Xiao, L. Zheng, T. Krishnamoorthi, I. King, S. Wang, P. Yin, J. J. Collins, Y. Li, Accurate RNA 3D structure prediction using a language model-based deep learning approach. *Nat. Methods* **21**, 2287–2298 (2024).

11. E. Morandi, I. Manfredonia, L. M. Simon, F. Anselmi, M. J. van Hemert, S. Oliviero, D. Incarnato, Genome-scale deconvolution of RNA structure ensembles. *Nat. Methods* **18**, 249–252 (2021).

12. P. J. Tomezsko, V. D. A. Corbin, P. Gupta, H. Swaminathan, M. Glasgow, S. Persad, M. D. Edwards, L. McIntosh, A. T. Papenfuss, A. Emery, R. Swanstrom, T. Zang, T. C. T. Lan, P. Bieniasz, D. R. Kuritzkes, A. Tsibris, S. Rouskin, Determination of RNA structural diversity and its role in HIV-1 RNA splicing. *Nature* **582**, 438–442 (2020).

13. S. W. Olson, A. W. Turner, J. W. Arney, I. Saleem, C. A. Weidmann, D. M. Margolis, K. M. Weeks, A. M. Mustoe, Discovery of a large-scale, cell-state-responsive allosteric switch in the 7SK RNA using DANCE-MaP. *Mol. Cell* **82**, 1708–1723.e10 (2022).

14. M. Yang, P. Zhu, J. Cheema, R. Bloomer, P. Mikulski, Q. Liu, Y. Zhang, C. Dean, Y. Ding, In vivo single-molecule analysis reveals COOLAIR RNA structural diversity. *Nature* **609**, 394–399 (2022).

15. P. P. Zarrinkar, J. R. Williamson, Kinetic intermediates in RNA folding. *Science* **265**, 918–924 (1994).

16. T. N. Wong, T. R. Sosnick, T. Pan, Folding of noncoding RNAs during transcription facilitated by pausing-induced nonnative structures. *Proc. Natl. Acad. Sci. U.S.A.* **104**, 17995–18000 (2007).

17. J. Blechar, V. de Jesus, B. Furtig, M. Hengesbach, H. Schwalbe, Shine-Dalgarno accessibility governs ribosome binding to the adenine riboswitch. *ACS Chem. Biol.* **19**, 607–618 (2024).

18. A. Haller, U. Rieder, M. Aigner, S. C. Blanchard, R. Micura, Conformational capture of the SAM-II riboswitch. *Nat. Chem. Biol.* **7**, 393–400 (2011).

19. A. J. Rinaldi, P. E. Lund, M. R. Blanco, N. G. Walter, The Shine-Dalgarno sequence of riboswitch-regulated single mRNAs shows ligand-dependent accessibility bursts. *Nat. Commun.* **7**, 8976 (2016).

20. M. F. Soulière, R. B. Altman, V. Schwarz, A. Haller, S. C. Blanchard, R. Micura, Tuning a riboswitch response through structural extension of a pseudoknot. *Proc. Natl. Acad. Sci. U.S.A.* **110**, E3256–E3264 (2013).

21. K. C. Suddala, J. Cabello-Villegas, M. Michnicka, C. Marshall, E. P. Nikonowicz, N. G. Walter, Hierarchical mechanism of amino acid sensing by the T-box riboswitch. *Nat. Commun.* **9**, 1896 (2018).

22. J. A. Hoerter, M. N. Lambert, M. J. Pereira, N. G. Walter, Dynamics inherent in helix 27 from *Escherichia coli* 16S ribosomal RNA. *Biochemistry* **43**, 14624–14636 (2004).

23. A. Chauvier, P. Ajmera, R. Yadav, N. G. Walter, Dynamic competition between a ligand and transcription factor NusA governs riboswitch-mediated transcription regulation. *Proc. Natl. Acad. Sci. U.S.A.* **118**, e2109026118 (2021).

24. O. Duss, G. A. Stepanyuk, A. Grot, S. E. O'Leary, J. D. Puglisi, J. R. Williamson, Real-time assembly of ribonucleoprotein complexes on nascent RNA transcripts. *Nat. Commun.* **9**, 5087 (2018).

25. O. Duss, G. A. Stepanyuk, J. D. Puglisi, J. R. Williamson, Transient protein-RNA interactions guide nascent ribosomal RNA folding. *Cell* **179**, 1357–1369.e16 (2019).

26. K. L. Frieda, S. M. Block, Direct observation of cotranscriptional folding in an adenine riboswitch. *Science* **338**, 397–400 (2012).

27. B. Hua, C. P. Jones, J. Mitra, P. J. Murray, R. Rosenthal, A. R. Ferre-D'Amare, T. Ha, Real-time monitoring of single ZTP riboswitches reveals a complex and kinetically controlled decision landscape. *Nat. Commun.* **11**, 4531 (2020).

28. M. L. Rodgers, S. A. Woodson, Transcription Increases the cooperativity of ribonucleoprotein assembly. *Cell* **179**, 1370–1381.e12 (2019).

29. H. Uhm, W. Kang, K. S. Ha, C. Kang, S. Hohng, Single-molecule FRET studies on the cotranscriptional folding of a thiamine pyrophosphate riboswitch. *Proc. Natl. Acad. Sci. U.S.A.* **115**, 331–336 (2018).

30. Z. Shajani, M. T. Sykes, J. R. Williamson, Assembly of bacterial ribosomes. *Annu. Rev. Biochem.* **80**, 501–526 (2011).

31. M. T. Sykes, J. R. Williamson, A complex assembly landscape for the 30S ribosomal subunit. *Annu. Rev. Biophys.* **38**, 197–215 (2009).

32. K. Gor, O. Duss, Emerging quantitative biochemical, structural, and biophysical methods for studying ribosome and protein-RNA complex assembly. *Biomolecules* **13**, 866 (2023).

33. S. A. Woodson, RNA folding and ribosome assembly. *Curr. Opin. Chem. Biol.* **12**, 667–673 (2008).

34. T. Powers, L. M. Changchien, G. R. Craven, H. F. Noller, Probing the assembly of the 3′ major domain of 16 S ribosomal RNA. Quaternary interactions involving ribosomal proteins S7, S9 and S19. *J. Mol. Biol.* **200**, 309–319 (1988).

35. T. Powers, H. F. Noller, Hydroxyl radical footprinting of ribosomal proteins on 16S rRNA. *RNA* **1**, 194–209 (1995).

36. T. Powers, S. Stern, L. M. Changchien, H. F. Noller, Probing the assembly of the 3′ major domain of 16 S rRNA. Interactions involving ribosomal proteins S2, S3, S10, S13 and S14. *J. Mol. Biol.* **201**, 697–716 (1988).

37. S. Stern, T. Powers, L. M. Changchien, H. F. Noller, RNA-protein interactions in 30S ribosomal subunits: Folding and function of 16S rRNA. *Science* **244**, 783–790 (1989).

38. W. A. Held, B. Ballou, S. Mizushima, M. Nomura, Assembly mapping of 30 S ribosomal proteins from *Escherichia coli*. Further studies. *J. Biol. Chem.* **249**, 3103–3111 (1974).

39. S. Mizushima, M. Nomura, Assembly mapping of 30S ribosomal proteins from *E. coli*. *Nature* **226**, 1214–1218 (1970).

40. T. Adilakshmi, D. L. Bellur, S. A. Woodson, Concurrent nucleation of 16S folding and induced fit in 30S ribosome assembly. *Nature* **455**, 1268–1272 (2008).

41. T. Adilakshmi, P. Ramaswamy, S. A. Woodson, Protein-independent folding pathway of the 16S rRNA 5′ domain. *J. Mol. Biol.* **351**, 508–519 (2005).

42. M. W. Talkington, G. Siuzdak, J. R. Williamson, An assembly landscape for the 30S ribosomal subunit. *Nature* **438**, 628–632 (2005).

43. H. Kim, S. C. Abeysirigunawarden, K. Chen, M. Mayerle, K. Ragunathan, Z. Luthey-Schulten, T. Ha, S. A. Woodson, Protein-guided RNA dynamics during early ribosome assembly. *Nature* **506**, 334–338 (2014).

44. A. M. Mulder, C. Yoshioka, A. H. Beck, A. E. Bunner, R. A. Milligan, C. S. Potter, B. Carragher, J. R. Williamson, Visualizing ribosome biogenesis: Parallel assembly pathways for the 30S subunit. *Science* **330**, 673–677 (2010).

45. M. L. Rodgers, Y. Sun, S. A. Woodson, Ribosomal protein S12 hastens nucleation of co-transcriptional ribosome assembly. *Biomolecules* **13**, 951 (2023).

46. I. I. Cisse, H. Kim, T. Ha, A rule of seven in Watson-Crick base-pairing of mismatched sequences. *Nat. Struct. Mol. Biol.* **19**, 623–627 (2012).

47. H. H. Lackey, Z. Chen, J. M. Harris, E. M. Peterson, J. M. Heemstra, Single-molecule kinetics show DNA pyrimidine content strongly affects RNA:DNA and TNA:DNA heteroduplex dissociation rates. *ACS Synth. Biol.* **9**, 249–253 (2020).

48. N. F. Dupuis, E. D. Holmstrom, D. J. Nesbitt, Single-molecule kinetics reveal cation-promoted DNA duplex formation through ordering of single-stranded helices. *Biophys. J.* **105**, 756–766 (2013).

49. T. Powers, G. Daubresse, H. F. Noller, Dynamics of in vitro assembly of 16 S rRNA into 30 S ribosomal subunits. *J. Mol. Biol.* **232**, 362–374 (1993).

50. A. Johnson-Buck, X. Su, M. D. Giraldez, M. Zhao, M. Tewari, N. G. Walter, Kinetic fingerprinting to identify and count single nucleic acids. *Nat. Biotechnol.* **33**, 730–732 (2015).

51. M. L. Rodgers, S. A. Woodson, A roadmap for rRNA folding and assembly during transcription. *Trends Biochem. Sci.* **46**, 889–901 (2021).

52. D. Herschlag, RNA chaperones and the RNA folding problem. *J. Biol. Chem.* **270**, 20871–20874 (1995).

53. D. Z. Bushhouse, E. K. Choi, L. M. Hertz, J. B. Lucks, How does RNA fold dynamically? *J. Mol. Biol.* **434**, 167665 (2022).

54. C. Weitzmann, S. J. Tumminia, M. Boublik, J. Ofengand, A paradigm for local conformational control of function in the ribosome: Binding of ribosomal protein S19 to *Escherichia coli* 16S rRNA in the presence of S7 is required for methylation of m2G966 and blocks methylation of m5C967 by their respective methyltransferases. *Nucleic Acids Res.* **19**, 7089–7095 (1991).

55. A. M. Popova, J. R. Williamson, Quantitative analysis of rRNA modifications using stable isotope labeling and mass spectrometry. *J. Am. Chem. Soc.* **136**, 2058–2069 (2014).

56. D. E. Burakovsky, I. V. Prokhorova, P. V. Sergiev, P. Milon, O. V. Sergeeva, A. A. Bogdanov, M. V. Rodnina, O. A. Dontsova, Impact of methylations of m2G966/m5C967 in 16S rRNA on bacterial fitness and translation initiation. *Nucleic Acids Res.* **40**, 7885–7895 (2012).

57. S. Rouskin, M. Zubradt, S. Washietl, M. Kellis, J. S. Weissman, Genome-wide probing of RNA structure reveals active unfolding of mRNA structures in vivo. *Nature* **505**, 701–705 (2014).

58. J. H. Davis, Y. Z. Tan, B. Carragher, C. S. Potter, D. Lyumkis, J. R. Williamson, Modular assembly of the bacterial large ribosomal subunit. *Cell* **167**, 1610–1622.e15 (2016).

59. J. L. Childs-Disney, R. Parkesh, M. Nakamori, C. A. Thornton, M. D. Disney, Rational design of bioactive, modularly assembled aminoglycosides targeting the RNA that causes myotonic dystrophy type 1. *ACS Chem. Biol.* **7**, 1984–1993 (2012).

60. S. Campagne, S. Boigner, S. Rüdisser, A. Moursy, L. Gillioz, A. Knörlein, J. Hall, H. Ratni, A. Cléry, F. H.-T. Allain, Structural basis of a small molecule targeting RNA for a specific splicing correction. *Nat. Chem. Biol.* **15**, 1191–1198 (2019).

61. J. L. Childs-Disney, X. Yang, Q. M. R. Gibaut, Y. Tong, R. T. Batey, M. D. Disney, Targeting RNA structures with small molecules. *Nat. Rev. Drug Discov.* **21**, 736–762 (2022).

62. R. Mehta, W. S. Champney, 30S ribosomal subunit assembly is a target for inhibition by aminoglycosides in *Escherichia coli*. *Antimicrob. Agents Chemother.* **46**, 1546–1549 (2002).

63. A. C. Stelzer, A. T. Frank, J. D. Kratz, M. D. Swanson, M. J. Gonzalez-Hernandez, J. Lee, I. Andricioaei, D. M. Markovitz, H. M. Al-Hashimi, Discovery of selective bioactive small molecules by targeting an RNA dynamic ensemble. *Nat. Chem. Biol.* **7**, 553–559 (2011).

64. F. P. Panei, P. Gkeka, M. Bonomi, Identifying small-molecules binding sites in RNA conformational ensembles with SHAMAN. *Nat. Commun.* **15**, 5725 (2024).

65. M. F. S. Degenhardt, H. F. Degenhardt, Y. R. Bhandari, Y. T. Lee, J. Ding, P. Yu, W. F. Heinz, J. R. Stagno, C. D. Schwieters, N. R. Watts, P. T. Wingfield, A. Rein, J. Zhang, Y. X. Wang, Determining structures of RNA conformers using AFM and deep neural networks. *Nature* **637**, 1234–1243 (2025).

66. Y. T. Lee, M. F. S. Degenhardt, I. Skeparnias, H. F. Degenhardt, Y. R. Bhandari, P. Yu, J. R. Stagno, L. Fan, J. Zhang, Y. X. Wang, The conformational space of RNase P RNA in solution. *Nature* **637**, 1244–1251 (2025).

67. C. R. Hofman, D. R. Corey, Targeting RNA with synthetic oligonucleotides: Clinical success invites new challenges. *Cell Chem. Biol.* **31**, 125–138 (2024).

68. B. H. Lloyd, R. V. Giles, D. G. Spiller, J. Grzybowski, D. M. Tidd, D. R. Sibson, Determination of optimal sites of antisense oligonucleotide cleavage within TNFalpha mRNA. *Nucleic Acids Res.* **29**, 3664–3673 (2001).

69. G. Hwang, M. Kwon, D. Seo, D. H. Kim, D. Lee, K. Lee, E. Kim, M. Kang, J.-H. Ryu, ASOptimizer: Optimizing antisense oligonucleotides through deep learning for IDO1 gene regulation. *Mol. Ther. Nucleic Acids* **35**, 102186 (2024).

70. A. Aartsma-Rus, A. Garanto, W. van Roon-Mom, E. M. McConnell, V. Suslovitch, W. X. Yan, J. K. Watts, T. W. Yu, Consensus guidelines for the design and in vitro preclinical efficacy testing N-of-1 exon skipping antisense oligonucleotides. *Nucleic Acid Ther.* **33**, 17–25 (2023).

71. Y. Shao, Y. Wu, C. Y. Chan, K. McDonough, Y. Ding, Rational design and rapid screening of antisense oligonucleotides for prokaryotic gene modulation. *Nucleic Acids Res.* **34**, 5660–5669 (2006).

72. M. Y. Chiang, H. Chan, M. A. Zounes, S. M. Freier, W. F. Lima, C. F. Bennett, Antisense oligonucleotides inhibit intercellular adhesion molecule 1 expression by two distinct mechanisms. *J. Biol. Chem.* **266**, 18162–18171 (1991).

73. B. P. Monia, J. F. Johnston, T. Geiger, M. Muller, D. Fabbro, Antitumor activity of a phosphorothioate antisense oligodeoxynucleotide targeted against C-raf kinase. *Nat. Med.* **2**, 668–675 (1996).

74. A. S. Alharbi, A. J. Garcin, K. A. Lennox, S. Pradeloux, C. Wong, S. Straub, R. Valentin, G. Pepin, H. M. Li, M. F. Nold, C. A. Nold-Petry, M. A. Behlke, M. P. Gantier, Rational design of antisense oligonucleotides modulating the activity of TLR7/8 agonists. *Nucleic Acids Res.* **48**, 7052–7065 (2020).

75. A. E. Bunner, A. H. Beck, J. R. Williamson, Kinetic cooperativity in *Escherichia coli* 30S ribosomal subunit reconstitution reveals additional complexity in the assembly landscape. *Proc. Natl. Acad. Sci. U.S.A.* **107**, 5417–5422 (2010).

76. S. S. Chen, J. R. Williamson, Characterization of the ribosome biogenesis landscape in *E. coli* using quantitative mass spectrometry. *J. Mol. Biol.* **425**, 767–779 (2013).

77. S. F. Clatterbuck Soper, R. P. Dator, P. A. Limbach, S. A. Woodson, In vivo X-ray footprinting of pre-30S ribosomes reveals chaperone-dependent remodeling of late assembly intermediates. *Mol. Cell* **52**, 506–516 (2013).

78. S. L. Bonilla, Q. Vicens, J. S. Kieft, Cryo-EM reveals an entangled kinetic trap in the folding of a catalytic RNA. *Sci. Adv.* **8**, eabq4144 (2022).

79. S. Li, M. Z. Palo, G. Pintilie, X. Zhang, Z. Su, K. Kappel, W. Chiu, K. Zhang, R. Das, Topological crossing in the misfolded *Tetrahymena* ribozyme resolved by cryo-EM. *Proc. Natl. Acad. Sci. U.S.A.* **119**, e2209146119 (2022).

80. J. Liu, E. K. S. McRae, M. Zhang, C. Geary, E. S. Andersen, G. Ren, Non-averaged single-molecule tertiary structures reveal RNA self-folding through individual-particle cryo-electron tomography. *Nat. Commun.* **15**, 9084 (2024).

81. D. K. Treiber, J. R. Williamson, Exposing the kinetic traps in RNA folding. *Curr. Opin. Struct. Biol.* **9**, 339–345 (1999).

82. D. K. Treiber, J. R. Williamson, Beyond kinetic traps in RNA folding. *Curr. Opin. Struct. Biol.* **11**, 309–314 (2001).

83. K. M. Weeks, T. R. Cech, Assembly of a ribonucleoprotein catalyst by tertiary structure capture. *Science* **271**, 345–348 (1996).

84. E. J. Strobel, L. Cheng, K. E. Berman, P. D. Carlson, J. B. Lucks, A ligand-gated strand displacement mechanism for ZTP riboswitch transcription control. *Nat. Chem. Biol.* **15**, 1067–1076 (2019).

85. K. E. Watters, E. J. Strobel, A. M. Yu, J. T. Lis, J. B. Lucks, Cotranscriptional folding of a riboswitch at nucleotide resolution. *Nat. Struct. Mol. Biol.* **23**, 1124–1131 (2016).

86. D. K. Treiber, M. S. Rook, P. P. Zarrinkar, J. R. Williamson, Kinetic intermediates trapped by native interactions in RNA folding. *Science* **279**, 1943–1946 (1998).

87. M. Kitagawa, T. Ara, M. Arifuzzaman, T. Ioka-Nakamichi, E. Inamoto, H. Toyonaga, H. Mori, Complete set of ORF clones of *Escherichia coli* ASKA library (a complete set of *E. coli* K-12 ORF archive): Unique resources for biological research. *DNA Res.* **12**, 291–299 (2005).

88. N. S. Qureshi, O. Duss, Tracking transcription-translation coupling in real time. *Nature* **637**, 487–495 (2024).

89. S. D. Chandradoss, A. C. Haagsma, Y. K. Lee, J. H. Hwang, J. M. Nam, C. Joo, Surface passivation for single-molecule protein studies. *J. Vis. Exp.*, 50549 (2014).

90. C. E. Aitken, R. A. Marshall, J. D. Puglisi, An oxygen scavenging system for improvement of dye stability in single-molecule fluorescence experiments. *Biophys. J.* **94**, 1826–1835 (2008).

91. I. Rasnik, S. A. McKinney, T. Ha, Nonblinking and long-lasting single-molecule fluorescence imaging. *Nat. Methods* **3**, 891–893 (2006).

92. M. F. Juette, D. S. Terry, M. R. Wasserman, R. B. Altman, Z. Zhou, H. Zhao, S. C. Blanchard, Single-molecule imaging of non-equilibrium molecular ensembles on the millisecond timescale. *Nat. Methods* **13**, 341–344 (2016).

93. J. Chen, R. V. Dalal, A. N. Petrov, A. Tsai, S. E. O'Leary, K. Chapin, J. Cheng, M. Ewan, P. L. Hsiung, P. Lundquist, S. W. Turner, D. R. Hsu, J. D. Puglisi, High-throughput platform for real-time monitoring of biological processes by multicolor single-molecule fluorescence. *Proc. Natl. Acad. Sci. U.S.A.* **111**, 664–669 (2014).

94. J. Chen, A. Petrov, A. Tsai, S. E. O'Leary, J. D. Puglisi, Coordinated conformational and compositional dynamics drive ribosome translocation. *Nat. Struct. Mol. Biol.* **20**, 718–727 (2013).

95. C. P. Lapointe, R. Grosely, M. Sokabe, C. Alvarado, J. Wang, E. Montabana, N. Villa, B. S. Shin, T. E. Dever, C. S. Fraser, I. S. Fernandez, J. D. Puglisi, eIF5B and eIF1A reorient initiator tRNA to allow ribosomal subunit joining. *Nature* **607**, 185–190 (2022).

96. S. H. Allen, K. P. Wong, The role of magnesium and potassium ions in the molecular mechanism of ribosome assembly: Hydrodynamic, conformational, and thermal stability studies of 16 S RNA from *Escherichia coli* ribosomes. *Arch. Biochem. Biophys.* **249**, 137–147 (1986).

97. A. Werner, Predicting translational diffusion of evolutionary conserved RNA structures by the nucleotide number. *Nucleic Acids Res.* **39**, e17 (2011).

98. M. Nomura, P. Traub, C. Guthrie, H. Nashimoto, The assembly of ribosomes. *J. Cell. Physiol.* **74** (Suppl. 1), 241–251 (1969).
